# Targeting the I7L Protease: A Rational Design for Anti-Monkeypox Drugs?

**DOI:** 10.3390/ijms24087119

**Published:** 2023-04-12

**Authors:** Andrea Dodaro, Matteo Pavan, Stefano Moro

**Affiliations:** Molecular Modeling Section (MMS), Department of Pharmaceutical and Pharmacological Sciences, University of Padova, Via Marzolo 5, 35131 Padova, Italy

**Keywords:** monkeypox virus, I7L protease, drug repurposing, DrugBank, virtual screening, homology modeling, AlphaFold, docking, protein–ligand interaction fingerprint, molecular dynamics

## Abstract

The latest monkeypox virus outbreak in 2022 showcased the potential threat of this viral zoonosis to public health. The lack of specific treatments against this infection and the success of viral protease inhibitors-based treatments against HIV, Hepatitis C, and SARS-CoV-2, brought the monkeypox virus I7L protease under the spotlight as a potential target for the development of specific and compelling drugs against this emerging disease. In the present work, the structure of the monkeypox virus I7L protease was modeled and thoroughly characterized through a dedicated computational study. Furthermore, structural information gathered in the first part of the study was exploited to virtually screen the DrugBank database, consisting of drugs approved by the Food and Drug Administration (FDA) and clinical-stage drug candidates, in search for readily repurposable compounds with similar binding features as TTP-6171, the only non-covalent I7L protease inhibitor reported in the literature. The virtual screening resulted in the identification of 14 potential inhibitors of the monkeypox I7L protease. Finally, based on data collected within the present work, some considerations on developing allosteric modulators of the I7L protease are reported.

## 1. Introduction

The 2022 monkeypox virus outbreak has spread all over the world with more than 86,000 confirmed cases to date, reaching 110 countries, 103 of which had not reported any previous cases [1,2]. Until recently, the monkeypox virus has been confined to the central-western part of Africa, where it is endemic, with seldom and limited cases of expansion to other continents [3]. Therefore, such an unprecedented worldwide outburst caused the World Health Organization (WHO) to declare it a Public Health Emergency of International Concern [4].

The monkeypox virus is an enveloped double-stranded DNA virus that belongs to the Orthopoxvirus genus of the Poxviridae family [5,6]. The vaccinia and variola viruses (the causative agent of smallpox) are also members of the Orthopox genus: meaning, they are immunologically cross-reactive and cross-protective. Indeed, thanks to it being less harmful to human health compared to the other members of the genus, the vaccinia virus has been used as a model to study the entire Orthopox genus [7]. Accordingly, in the present work, molecular targets will be referred to after the vaccinia virus nomenclature.

Nevertheless, the clinical manifestation of the monkeypox infection resembles the ones of smallpox, albeit milder [8]. Indeed, the most typical symptom is a febrile rush [8], usually accompanied by the emergence of umbilicated skin lesions [9], principally located on the extremities and the face [6], which can cause several severe medical complications, which may ultimately result in the patient’s death [8], with a maximum lethality rate of 4–10%, depending on the strain in the countries where this virus is endemic [10].

Despite being mainly transmitted through direct contact with infected animals’ bodily fluids and flesh, this virus can also be spread among humans via bodily fluid exchanges and prolonged face-to-face exposure that can convey the virus through respiratory droplets [11].

To date, no monkeypox virus-specific drug has been designed, with vaccination being proposed as a treatment against this pathogen owing to its long incubation time (2–3 weeks) [12]. Another recently proposed treatment is Tecovirimat, a drug that blocks the viral protein VP37, which has been approved by the Food and Drug Administration for the treatment of smallpox [12,13].

The potential for rapid human-to-human diffusion of this virus demonstrated by the 2022 outbreak and the lack of effective and specific treatments demand the rapid identification and development of novel pharmacological tools to manage this disease. 

According to the literature, within the replication cycle of the model vaccinia virus, a fundamental step is the maturation of non-infective virions from immature intracellular virions (IV) to mature intracellular virions (IMV). This maturation process is linked to the activity of the viral protease I7L, which is responsible for the cleavage of A17, a structural protein involved in the formation of the crescent membrane that eventually leads to the virion assembly. This cysteine protease is also responsible for the cleavage of several core proteins, including P25K/VP8, P4a, and P4b, which form more than 30% of the mass of the virion [14,15].

Owing to its pivotal role within the replication cycle of Orthopox viruses, and to the good results of drug discovery campaigns resulting in the development of protease inhibitors against HIV, Hepatitis C, and SARS-CoV-2 [16], the I7L protease is a promising target for the design of effective and specific drugs against the monkeypox virus, and as such, it has been selected as the focus of the present computational study. Particularly, the 99% amino acid identity between vaccinia, variola, and monkeypox orthologues [17,18], allows for extending considerations retrieved from the characterization of this protease to the whole genus, paving the way for the development of compounds with pan-anti-poxviral activity.

Since the structure of the I7L protease has not been experimentally determined yet, in the present work it was modeled and characterized through the combination of various computer-aided drug discovery (CADD) methods, including homology modeling, de novo prediction, in silico mutagenesis, and molecular dynamics. Furthermore, the acquired structural insights were exploited to perform a docking-based virtual screening on the DrugBank database, a library of more than 10,000 compounds, including drugs approved by the Food and Drug Administration, and clinical-stage drug candidates, seeking ligands with binding features comparable with the ones of TTP-6171, the only noncovalent inhibitor of the I7L protease reported in the literature, which could be readily repurposed against the monkeypox virus.

## 2. Results

### 2.1. Modeling the I7L Protease Structure

Since the structure of the monkeypox I7L protease has not been experimentally solved yet, three different models were created, either through homology modeling or de novo prediction.

Based on two previously published works that led to the structure-based identification of both covalent and noncovalent inhibitors of the vaccinia virus I7L protease [18,19], a first model was built through the Phyre2 [20] server using the Saccharomyces cerevisiae Ulp-1 protease (PDB ID: 1EUV) as a template. 

**Figure 1:**
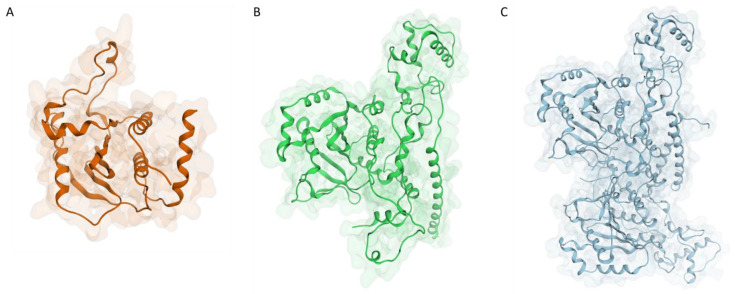
Structure of the three models generated for the monkeypox I7L protease: (**A**) monomeric model generated with Phyre2 using S. cerevisiae Ulp-1 protease (PDB ID: 1EUV) as a template; (**B**) monomeric model generated with AlphaFold2; (**C**) dimeric model generated with AlphaFold2.

A second model was obtained through ColabFold [21], a web-based front-end for protein structure fold prediction using AlphaFold2 [22,23]. Finally, since it has been reported in the literature how homodimerization can influence the catalytic activity of the I7L protease [24], a third model representing the protein in its dimeric form was generated through ColabFold. The structures of the three different models are reported in Figure 1.

**Figure 2:**
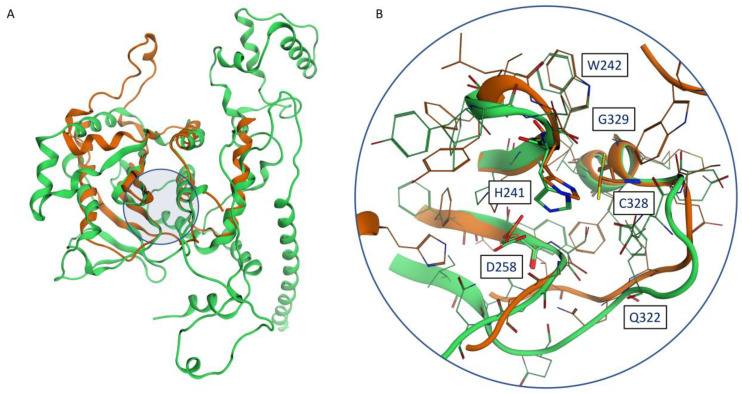
(**A**) Superposition between the Phyre2 monomeric model (orange) and the AlphaFold2 model (green): the location of the catalytic site is highlighted by a blue circle. (**B**) Superposition between the catalytic sites of the Phyre2 monomeric model (orange) and the AlphaFold2 model (green): conserved residues reported in the work of Byrd et al. [17] are labeled.

Although the two monomeric models look quite different at a first glance, a superposition between the two reveals how differences are mainly located away from the catalytic site, which is, for the most part, quite conserved and superimposable (Figure 2). 

**Figure 3:**
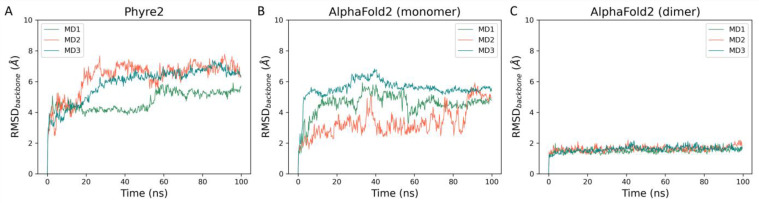
This panel showcases the time-dependent evolution of the backbone RMSD calculated throughout molecular dynamics simulations performed on each of the three I7L protease models: (**A**) Phyre2 model (monomer), (**B**) AlphaFold2 model (monomer), and (**C**) AlphaFold2 model (dimer).

To assess the validity of generated models, we performed molecular dynamics simulations to evaluate the models’ stability. Three independent MD replicates, with each 100 ns in duration, were carried out on each of the models. The time-dependent evolution of the backbone RMSD for each of the models is reported in Figure 3.

As can be noticed in Figure 3, there is a net difference in backbone stability among the three generated models. Particularly, the two monomeric models, which are much less stable than the dimeric one, generated by AlphaFold2, as highlighted not only by the high RMSD values reached at the beginning of the simulation but also by the wide fluctuations throughout the whole length of the trajectory. The low stability of the Phyre2 model can be explained by the fact that a good portion of the protease structure is missing, specifically the N-term (residues 1–165) and the C-term (365–423) since they did not have a correspondent residue in the template structure. 

The difference in stability between the two AlphaFold2 models can instead be explained by the fact that homodimerization seems to enhance enzymatic activity [24] and that the N-terminal truncated protease (229–423) is not catalytically competent [17]. Indeed, as can be observed in Figure S1 panel A (Supplementary Materials), the interface between the two monomers in the AlphaFold2 model largely involves the N-terminal region, including the G29-LCSNIDV-S37 loop, which has been flagged as crucial for the regulation of the catalytic activity of I7L [25]. The stabilization of the N-terminal region within the dimer model is also highlighted by the per-residue RMSF derived from the molecular dynamics simulations (Figure S1, panels B and C, Supplementary Materials). Furthermore, the secondary structure of the protease is well conserved throughout the MD simulations of the dimer model, as highlighted by the plots reported in Figure S2 (Supplementary Materials). Taken together, these observations indicate the dimeric AlphaFold2 model as the most accurate representation of the monkeypox virus I7L protease.

To further validate our protease model, we performed in silico mutagenesis on each conserved residue indicated in the work by Byrd et al. [17]. Specifically, a virtual alanine scanning was executed through the appropriate module of the Molecular Operating Environment suite (MOE) for all mutants. Coherently with experimental data, all investigated mutations are predicted to destabilize the I7L protease (Table S1, Supplementary Materials). Intriguingly, in the original work, the D258A mutant is determined to be viable, despite D258 being involved in a hydrogen bond with the catalytic H241, which mostly assists the catalysis process by ensuring the right orientation of the catalytic histidine towards C328 [26]. Traditionally, cysteine proteases, including SARS-CoV-2 3CL^pro^ [27], have been associated with catalytic dyads [28], while catalytic triads have been linked to serine proteases, due to the different nucleophilicity profile between thiolate and alkoxide ions [26] However, based on the sequence conservation around the catalytic site between the I7L protease and the yeast Ulp-1 protease [29], a cysteine protease characterized by an Asp-His-Cys catalytic triad [30], and on the presence of a catalytic triad involving either and Asp, Asn or a Glu in other proteases belonging to the CE clan [31,32], such as the human adenovirus 2 proteinase (AVP) [33] and African swine fever virus pS273R protease [34], we can assume that the proposed fold is plausible and that the conserved catalytic activity of the D258A mutant could be attributed to compensatory effects, such as homodimerization and the presence of DNA/RNA [24]. Indeed, the catalytic activity of the prototypical CE protease AVP is greatly increased by the peptide cofactor pVIc and the presence of DNA [35], with the pVIc peptide occupying a similar interaction interface as the monomer–monomer one in our dimeric model [36] (Figure S3, Supplementary Material). Considering that the binding to the pVIc cofactor induces a conformational change of the active site [36], it is possible that the instability noticed in MD simulations of our AlphaFold2 monomeric model is related to the absence of the stabilizing/allosteric effect of the second monomer. 

In the case of the pVIc peptide, the interaction is driven by the formation of a disulfide bond between two conserved cysteine residues, namely C10 (pVIc) and C104 (AVP) [37,38]. Intriguingly, in our dimeric model, two cysteine residues located in the C-terminal alpha helix (C408) face each other (Figure S4, Supplementary Materials), suggesting the possible formation of a disulfide bond that could stabilize the dimeric form of the protease, hence, increasing its catalytic activity. 

Although the vicinity of these two residues may be an AlphaFold2 artifact, the good confidence in AlphaFold2′s prediction of the C-terminal helix highlighted by the high pLDDT value (Figure S5, Supplementary Materials) and the observation that, throughout our MD simulations, the distance between the two cysteines remains stable and compatible with the formation of a disulfide bond [39] (Table S2, Supplementary Materials), reinforce the plausibility of this structural hypothesis. Intriguingly, regions associated with the highest uncertainty in the prediction are located at the interface between the two monomers in the dimeric model, coherently with the hypothesis of a stabilizing role of the catalytic domain carried out by the homodimerization process.

Furthermore, the Q322A and D248A mutants were also characterized through MD simulations. We decided to focus on these two mutations since the other ones were readily explainable by looking at the modeled three-dimensional structure of the protease. For instance, H241, C328, and D258 are all part of the catalytic triad. G329, instead, is the residue that follows the catalytic cysteine residue, and it has been demonstrated how the protease has substrate specificity for residues bearing small sidechains, such as glycine and alanine [24]. Likewise, W242 is a core residue of the binding site, that most likely concurs in defining the substrate specificity towards small sidechain residues straddling across the cleavage site [18], as in the case of Ulp-1 and other SUMO proteases [40]. Indeed, within our MD simulations, two different clusters related to W242 conformation can be observed: in the “closed” conformation, the tryptophane sidechain occupies the subpocket usually occupied by the P1 glycine residue of the substrate, while in the “open” conformation the tryptophane sidechain forms a tunnel that allows the harboring of the substrate near to the catalytic cysteine (Figure S6, Supplementary Materials).

In the AlphaFold2 model, Q322 is involved in a network of interactions with the backbone of L324 and the sidechain of S259, consistently with a possible crucial role in the stabilization of the postcleavage subsite and the formation of the oxyanion hole [19] (Figure S7, panel A Supplementary Materials). Likewise, D248, which has been flagged as a pivotal residue for the catalytic activity of the protease despite not being part of the triad or the catalytic site, is predicted to be involved in another crucial network of interactions with the backbone of K250, N251, and, most notably, the sidechain of R230 (Figure S7, panel B Supplementary Materials). As expected, coherently with the mutagenesis study performed with MOE, the energetic analyses performed on MD trajectories carried out on both the mutants and the wildtype I7L protease reveal how both the Q322A and D248A mutants have an unfavorable energetic profile compared to the wildtype protein, although the reduced timescale of our MD simulations did not allow for harsh local alterations of the protein fold (Figures S8 and S9, Supplementary Materials). 

Finally, to further characterize the interactive features of the catalytic site, we modeled the binding mode of four substrate peptide sequences reported in the literature, namely P4a (G614-S615), P4b (G61-A62), and two different cleavage sites on VP8/P25K (G18-S19 and G32-A33, respectively) [24,41]. Although the estimation of the cleavage rate of those substrate peptides slightly varies depending on the experimental conditions, it is possible to assume that both VP8 [17,24,25,41] and P4b [24,25,41] are cleaved faster than P4a and that VP8 is more rapidly processed than P4b [24,41]. Coherently, our modeling indicates how both VP8 (G32-A33) and P4b have better interaction profiles with the catalytic site compared to the VP8 (G18-S19) and P4a (Figures S10–S13 and Table S3, Supplementary Materials). 

Furthermore, based on our structural analysis, it can be noticed how peptides with higher cleavage rates have better electrostatic complementarities with the binding groove, especially because of the presence of the negatively charged residues in positions P7–P8, which have been previously linked with substrate specificity [24], and which interacts with an electropositive patch formed by basic residues, such as R124, R172, and R196 (Figure S14, Supplementary Materials). Consequently, it is possible to hypothesize that the faster cleavage rates of AGA sequences compared to AGS [41] may not be related to the P1′ residue, coherently with the tolerated variability in P2–P1′ residues across substrate sequences within the CE clan [42], yet might be instead related to ancillary residues located before and after the proteolytic cut site, which confer an increased affinity towards the binding groove. 

### 2.2. Virtual Screening of the DrugBank Database

**Figure 4:**
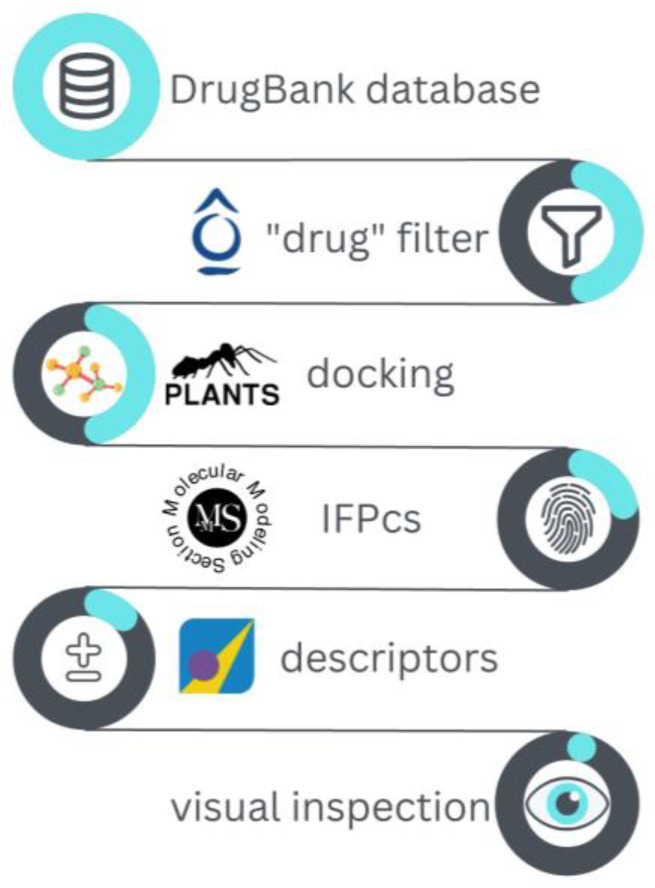
Schematic representation of the workflow for the virtual screening performed on the DrugBank database against the monkeypox virus I7L protease catalytic site.

The validated I7L protease structure model was used to perform a docking-based virtual screening of the DrugBank database toward the potential repurposing of existing drugs as antiviral agents against the monkeypox virus. Specifically, DrugBank is a library that collects about 11,614 molecules, including FDA-approved drugs (both small molecules and biotech), nutraceuticals, and experimental therapeutics (molecules that reached various stages of clinical experimentation). The workflow for the executed virtual screening is schematized in Figure 4 and explained in detail hereafter.

The library was docked to the I7L protease binding site (centered around C328, one of the members of the catalytic triad) using the PLANTS docking program, generating 10 poses for each ligand. The only noncovalent I7L protease inhibitor available in the literature, TTP-6171, was also included in the library as a positive control. 

**Figure 5:**
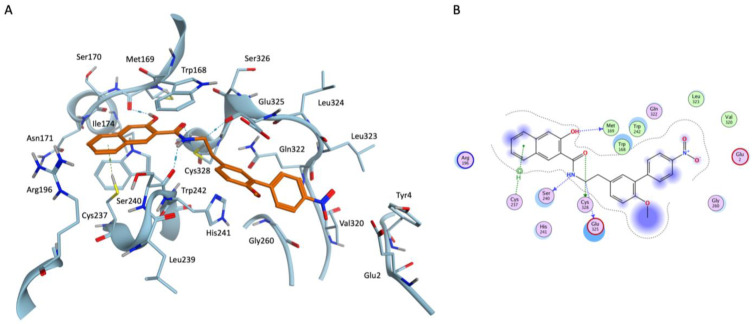
(**A**) Three-dimensional representation for the docking-predicted binding mode for the reference compound TTP-6171. (**B**) Bidimensional representation for the docking-predicted binding mode for the reference compound TTP-6171.

At first, docking poses for the query compounds were compared to the selected TTP-6171 pose reported in Figure 5, chosen among the top-scoring poses through visual inspection. In this docking pose the compound nicely complements the shape of the binding site and its key interaction features. Particularly, in this pose the naphthol moiety of TTP-6171 stacks between the sidechains of W168 and W242, establishing both hydrophobic and π-stacking interactions. Furthermore, the central node of the ligand (that mimics a glycine residue, which is commonly found at the cleavage site), is placed in the narrowest portion of the channel, close to the catalytic C328. Finally, further stabilizing interactions include the H-bond between the backbone of M169 and the hydroxyl group of the naphthol and another H-bond between the amide hydrogen of the “pseudo-glycine” portion of the ligand and the backbone of S240.

The comparison between the reference and the query compounds was carried out through the recently developed IFP_CS_ scoring function [43], to retain only those poses that presented an interaction pattern congruent with the one for the reference. Practically, both the reference pose and the queries were converted into protein–ligand interaction fingerprints via the appropriate function of the Open Drug Discovery Toolkit library, and compared through the cosine similarity metrics, keeping only those poses that matched most of the binding features modeled for the TTP-6171.

Afterward, the remaining poses were further filtered based on some descriptors provided by the Molecular Operating Environment suite. Particularly, the van der Waals and electrostatic contributions to the interaction energy were used to keep only those poses with a similar energetic contribution as the reference pose for the TTP-6171. Furthermore, due to the shallow and solvent-exposed nature of the binding site, a cutoff on the percentage of solvent-exposed ligand surface was used to keep only those poses that presented a similar level of shielding from the solvent as the TTP-6171. Finally, a round of visual inspection of poses was carried out, to prioritize poses with the best shape complementarity with the binding site, resulting in the selection of 14 compounds. The docking poses for the 14 selected compounds are reported in Figures S15–S28 and Video S1 (Supplementary Materials), while detailed information about each compound can be found in Table S4 (Supplementary Materials). 

**Figure 6:**
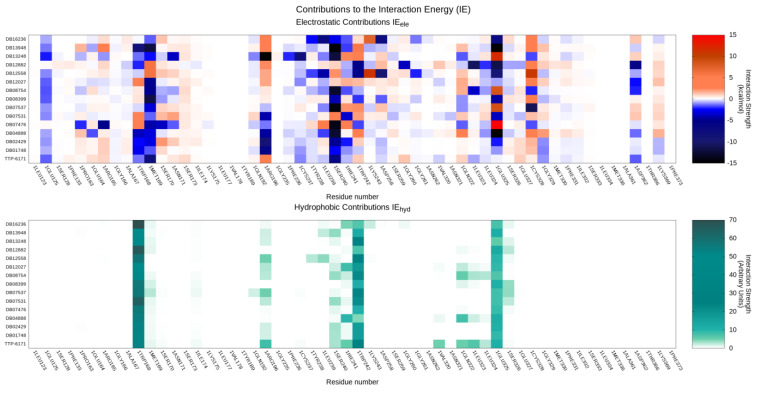
Per-residue decomposition of the main contributions to the protein-ligand interaction energy for both the reference TTP-6171 and query compounds identified through the docking-based virtual screening: the upper panel highlights electrostatic interactions, while the lower panel showcases hydrophobic interactions.

The conservation of crucial binding interactions between the reference TTP-6171 and query compounds found through the docking-based virtual screening is highlighted by the aggregate heatmap, reported in Figure 6, which reports a per-residue interaction energy decomposition for both the electrostatic and hydrophobic components to the protein–ligand interaction.

As expected by the selection process based on the similarity of the protein–ligand interaction fingerprints, interactions with key residues composing the binding site regarding both the electrostatic and hydrophobic contributions to the interaction energy are conserved through the series, despite differences in the chemical structure of the ligands. Particularly, our selected compounds are predicted to have good electrostatic interactions with E125, W168, M169, and S240, while main hydrophobic contributions can be principally traced to W168, W242, and the alkyl chain of E125.

To cope with the neglected solvent effect and the static nature of molecular docking, a short MD-based post-docking refinement was carried out. Principal descriptors extracted from the post-docking refinement are summarized in Table 1. 

As can be observed in Table 1, docking poses for the 14 selected compounds show comparable stability and quality of interaction to the reference TTP-6171 pose, except for DB13248, which shows a relatively high RMSF value, indicative of an unstable pose. Interestingly, this ligand presents the lowest hydrophobic contribution to the interaction energy, supporting the previously stated hypothesis of a hydrophobic-driven binding at the catalytic site.

## 3. Discussion

The 2022 monkeypox virus outbreak brought to the attention of the general audience this relatively unconsidered pathogen as a potential threat to public health. To mitigate the lack of effective and specific treatment for this virus, and in consideration of the success of recent drug discovery campaigns for the design of viral protease inhibitors, we performed a preliminary computational study to structurally characterize the I7L protease and identify potentially repurposable drugs as pharmacological tools against this disease.

Three different I7L models were generated, either through homology modeling or de novo prediction, describing both the monomeric and dimeric forms of the protease. MD simulations performed on generated models highlighted a net difference in stability between the two monomeric models and the dimeric one, in favor of the latter. This difference is coherent with the fact that homodimerization enhances the catalytic activity of I7L [24]. Furthermore, the proposed dimerization interface is coherent with a similar allosteric effect portrayed on the AVP protease, belonging to the same CE clan as the I7L, by the peptide cofactor pVIc [36,37], and with the observation of the importance of the N-terminal region of I7L for the catalytic activity [17,25], validating the reliability of the proposed model. 

After the model validation, a docking-based virtual screening was carried out on the DrugBank database, a library containing FDA-approved drugs and drug candidates in various stages of clinical experimentation, seeking readily repurposable compounds as antiviral agents against the monkeypox virus. 

At first, the binding mode of TTP-6171, the only non-covalent I7L inhibitor reported in the literature, was elucidated. This compound is predicted to nicely fit into the catalytic groove, owing to the good shape complementarity with the pocket. From an interaction perspective, this compound is predicted to insert a naphthol moiety amid two conserved tryptophane residues, namely W168 and W242. Based on structural data on similar proteases [33,34,44], mutagenesis data on the pivotal role of these two aromatic residues [17], and the observation of equilibrium between an open and a closed conformation of the catalytic tunnel regulated by the relative position of these two tryptophane residues throughout our MD simulations, it seems reasonable to mark this as a crucial interaction in explaining the inhibitory activity of TTP-6171. Furthermore, this ligand placement allows for establishing other ancillary stabilizing interactions with the pocket, other than placing a “pseudo-glycine” ligand moiety towards the catalytic C328, as happens with peptide substrate sequences. 

Intriguingly, two previously published computational studies by Lam et al. [45] and Dubey et al. [46] both predicted different binding modes for TTP-6171, despite working on a superimposable I7L protease model generated through AlphaFold2. Particularly, Dubey et al. described a binding mode for TTP-6171, which involves interactions with residues such as C22, H23, S26, L27, N33, V36, L40, I371, Y393, K394, and E397. This pocket is located on the opposite side of the catalytic site and includes the G29–S37 loop, which is involved in the regulation of catalytic activity, either through homodimerization or interaction with nucleic acids [17,24,25]. In our dimeric model, this subsidiary pocket is predicted to harbor the C-terminal α-helix of the second monomer. Although this putative mechanism of action would theoretically explain the TTP-6171 activity, there are some concerns related to this prediction. First, this pose is not in agreement with data on TTP-6171-resistant mutants (Y104C and L324M), which are both located far away from this binding pocket [19]. Moreover, the monomeric protease model used in their study undergoes a notable conformational rearrangement during molecular dynamics simulations, as well as the predicted binding pose for TTP-6171, making it difficult to assess if the instability observed for their pose is related to the protein instability or the poor quality of interaction between the ligand and the protein. 

In the work of Lam et al. [45], instead, TTP-6171 is predicted to bind in a solvent-exposed cleft situated right below the cleavage site. Although this prediction is more coherent with data on compound-resistant mutants (the L324M mutation is located on the oxyanion loop that borders the catalytic site), there are some questions about its validity. First, the chemical structure of TTP-6171 shown in their work is not coherent with the one reported in the original work by Byrd et al. [19], which was instead utilized in this work and the one in Dubey et al. Furthermore, in their post-docking refinement, the proposed binding mode is not stable, rapidly reaching an alternative, extended conformation (RMSD = 12 Å), which is, then, maintained for the rest of the simulation. In virtue of the shallow and solvent-exposed nature of the catalytic site, which does not offer many anchoring points for small molecule inhibitors, and the comparably higher stability of the docking poses presented for their virtual screening hits (which straddle across the catalytic cleft in a similar way to docking poses for our virtual hits) to the one of TTP-6171, it is possible to assume that our binding mode is more convincing. Another piece of evidence supporting this working hypothesis is that substrate peptides binding seems to be more driven by residues located at the N-terminus end of the peptide rather than the ones located at the C-terminus end of the cleavage sites, coherently with a certain tolerance and variability like post-cleavage residues [17,24,25,41], and with the solvent-exposed nature of the lower cleft. Finally, in the work by Katritch et al. [18], which identified 97 covalent inhibitors of the I7L protease through a docking-based virtual screening executed on a homology model based on the yeast Ulp-1 protease, the modeled binding modes presented for the hit compounds closely resembled the one proposed in the present work for TTP-6171 and the noncovalent hits extracted from our virtual screening.

Although the strategy of covalently targeting the catalytic site seems advantageous, considering the difficulties of targeting such shallow and solvent-exposed binding grooves, without a rational design supporting the idea of a targeted covalent inhibition, the lack of selectivity and potential associated adverse effect could hamper the development of the initial hits into pharmacological tools with any real-world application [47], hence, the idea of performing a structure-based virtual screening for identifying noncovalent binders. Specifically, we focused our attention on the DrugBank database, a library composed of FDA-approved drugs and clinical drug candidates, since the identification of well-characterized compounds from a pharmacokinetic and pharmacological perspective would drastically reduce the time needed for transitioning a hit into a candidate [48].

Our multistage virtual screening led to the selection of 14 candidate inhibitors of the I7L protease. Compared to the reference TTP-6171, the selected compounds present similar shapes and electrostatic features regardless of their chemical scaffold, other than presenting a superimposable predicted binding pattern. Unfortunately, as reported in Table S4 (Supplementary Materials), only two out of fourteen virtual hits belong to the “approved” ensemble of the DrugBank database (Mitapivat, a pyruvate kinase activator used for hemolytic anemia, and Phthalylsulfathiazole, which is employed as a gastrointestinal antibiotic), with the other four compounds belonging to the “investigational” group and the rest being part of the “experimental” group. Furthermore, many of the “investigational” or “experimental” hits have been developed as antitumoral agents, thus, preventing their application in the treatment of a viral disease due to the intolerable adverse effects. However, if their activity should be experimentally confirmed, they could serve as starting point for the development of specifically designed antiviral compounds, other than validating the usefulness of the presented structural model of the I7L protease and similar models built by homology with the yeast Ulp-1 protease. 

Finally, due to the peculiar topological features of the catalytic site, it might be worth investigating alternative strategies for modulating I7L proteolytic activity that does not involve competitive binding within the catalytic groove. According to our structural investigation, indeed, three possible pathways could be pursued: targeting the electropositive exosite defined by basic residues, such as R124, R172, and R196 (exosite 1), aiming at the allosteric pocket flanking the N29–S37 loop (exosite 2), or targeting the dimerization interface. Due to the different nature of those hotspots, with exosite 2 seemingly being the only druggable cavity through a small molecule inhibitor, the development of aptamers could represent a way to produce potent and selective binding at those flat and solvent-exposed surfaces, based on previous attempts on similar targets, including the human thrombin [49].

Despite the aforementioned issues, developing I7L protease inhibitors represents a promising strategy complementary or alternative to addressing other potentially viable targets. For example, despite the recent release of the experimentally determined structure of the F8 DNA polymerase [50], which provides a structural basis for the rational development of compounds able to inhibit the synthesis of viral DNA, targeting this macromolecular machinery is complicated by pharmacodynamic issues, such as an intrinsic lack of selectivity of nucleoside analogs, and pharmacokinetic limitations, such as their conversion to the triphosphate form [51]. Another possible route would be to target the membrane protein VP37, the molecular target of Tecovirimat, although the lack of experimentally solved structure of this protein and atomistic details on the mechanism of its inhibition would present similar challenges to those described for the I7L protease [52].

Lastly, it is important to stress that although the present work has been executed using state-of-the-art CADD methodologies and through systematic comparison with available experimental evidence, it remains a preliminary theoretical study that needs further experimental validation, both from a structural and biological perspective. Indeed, although the present work provided some plausible mechanistic explanations on previously published data, the low amount of information available and the emergence of novel structural insights, such as the possible formation of a disulfide bridge that regulates the dimerization equilibrium will hopefully inspire some targeted biological investigations that will complement the data reported in the present work. 

## 4. Materials and Methods

### 4.1. Hardware Overview

Each ordinary molecular modeling procedure was carried out on a Linux Workstation, shipping a 12-core Intel(R) Xeon(R) CPU E5-1650. The docking-based virtual screening was performed on a 64-core AMD Opteron™ Processor 6376 CPU cluster. Molecular dynamics simulations were conducted on a GPU cluster composed of 20 NVIDIA devices ranging from GTX980 to RTX2080Ti. Each machine used Ubuntu 20.04 as its operating system. 

### 4.2. I7L Protease Structure Modeling

The three-dimensional structure of the monkeypox virus I7L protease has not been experimentally determined yet. For this reason, three different models were created, either through homology modeling or de novo prediction.

Initially, the primary sequence of the I7L protease was retrieved from the UniProt [53] database (accession code: Q5IXV7). The Protein-BLAST [54,55] webserver was used to search for suitable templates for the generation of a homology model, leading to the identification of the C-terminal domain of the ULP-1 protease of Saccharomyces cerevisiae (PDB ID: 1EUV [44]) as the best possible template (17% sequence identity with the monkeypox virus I7L protease). Considering that this structure was already used in previous scientific works for the generation of a homology model of the vaccinia virus I7L protease that led to the identification of the noncovalent inhibitor TTP-6171 [19] and 97 covalent inhibitors [18], the high sequence identity of the I7L gene among the Orthopoxviridae family (between 95 to 99%), and the complete conservation of catalytic site residues [19], a first model of the monkeypox I7L protease in its monomeric form was constructed through the Phyre2 [20] webserver, using structure 1EUV as a template.

A second model of the I7L protease structure in its monomeric form was obtained through ColabFold [21] 1.3, a web-accessible front-end for de novo prediction of protein structures through AlphaFold [22,23] 2.2. The same method was used for generating a third model of the I7L protease in its dimeric form since it has been reported how homodimerization can influence its catalytic activity [24]. 

### 4.3. Models Validation

To assess the reliability of the three models generated as presented in Section 4.2, their stability was evaluated through a series of classic molecular dynamics simulations. At first, each model was checked using the “Structure Preparation” tool from the Molecular Operating Environment (MOE [56]) 2022.02 suite. Afterward, the “Protonate3D” tool was exploited to assign the most probable protonation state to each residue (pH 7.4, T = 310 K, i.f. = 0.154). Finally, partial charges were attributed according to the AMBER10:EHT force field, as implemented in MOE. 

The system setup for MD simulation of preprocessed structures was, then, carried out by combining different packages from Visual Molecular Dynamics (VMD [57]) 1.9.2 and the AmberTools22 [58,59] suite. Specifically, parameters for protein atoms, water molecules, and ions were assigned according to the ff14SB [60] force field. 

Each protease model was solvated in a rectangular base prism box of TIP3P [61] water molecules, ensuring a minimum 15 Å distance between each protein atom and the box border. Furthermore, the appropriate number of sodium and chlorine ions were added to electroneutralize the box and reach a salt concentration of 0.154 M. Before MD simulation, 500 steps of energy minimization with the conjugate-gradient algorithm were employed to remove clashes within each system. 

MD simulations presented in the current article were carried out using the ACEMD [62] 3.5 engine, which is based on the open-source Python library for molecular simulations OpenMM [63] Specifically, a 2 fs integration timestep was used, the M-SHAKE algorithm was used to constrain the length of bonds involving hydrogen atoms, the particle-mesh Ewald [64] method with cubic spline interpolation, and 1 Å grid spacing were used to compute electrostatic interactions, while a 9.0 Å cutoff was applied for the calculation of Lennard-Jones interactions. Before the productive simulations, a two-stage equilibration process was carried out. In the first equilibration step, 0.1 ns of NVT simulation were performed, with harmonic positional restraints imposed on each protein atom, leaving water molecules and ions unconstrained. In the second equilibration run, 0.5 ns of NPT simulation were carried out, with the same harmonic positional restraints applied only on the protein backbone. In both cases, a 5 kcal mol^−1^ Å^−2^ force constant was applied on restrained atoms for the whole length of the simulation. For each simulation, the temperature was maintained at 310 K through a Langevin thermostat [65], while for simulations in the NPT ensemble, the pressure was maintained at 1 atm through a Monte Carlo barostat [66]. For each investigated model, three 100 ns independent MD replicates were performed in the NVT ensemble (T = 310 K). Then, trajectories were analyzed by calculating the time-dependent evolution of the backbone RMSD and the protein secondary structure, other than the per-residue RMSF, making use of the appropriate functions of the MDA analysis [67,68], Python library, and VMD. Finally, representative frames were extracted from each trajectory through clustering with the TTCLUST [69] Python package. 

### 4.4. In Silico Mutagenesis

To further assess the validity of the dimeric protease model, mutants were generated in silico based on previously published experimental data [17] and evaluated through the same MD-based protocol reported in Section 4.2. Mutants were prepared through the editing of the dimeric protease model with the “alanine scanning” tool in MOE 2022.02, calculating the stability difference between the mutated protein and the wildtype expressed as dStability (kcal/mol). Furthermore, based on visual inspection of the protease model, Q322A and D248A were further evaluated using the previously described MD-based protocol, resulting in three independent 100 ns MD replicates for each case.

### 4.5. Substrate Peptides Modeling

The sequences for each of the four modeled substrate peptides (P9-P4′), namely P4a, P4b, and the two cleavage sites on VP8, were retrieved from the work of Aleshin et al. [24]. Peptide binding poses were manually modeled by templating the P2–P1 positions from the peptide substrate of yeast Ulp-1 protease (PDB ID: 1EUV) and iteratively adding missing residues through the combination of the “Protein Builder” tool in MOE 2022.02, and cycles of energy minimization, according to the AMBER10:EHT force field, as implemented in MOE 2022.02. The obtained complexes were relaxed through 3 independent, 3 ns MD replicates, using the same procedures described in Section 4.2. An approximation of the binding free energy was obtained through the MM/GBSA method, as implemented in the AmberTools22. The last trajectory frame extracted from the trajectory associated with the closest-to-average binding free energy value was chosen as representative and visually analyzed, while a per-residue interaction energy matrix calculated on the representative trajectory was obtained through the github.com/molecularmodelingsection/SuMD-analyzer Python script presented and discussed in the work by Pavan et al. [49].

### 4.6. Virtual Library Preparation

The complete DrugBank 5.1.9 database was retrieved from the DrugBank website and prepared for docking calculations using various tools from the QUACPAC OpenEye suite [70] and CORINA Classic [71], as follows. At first, the “filter” tool was used to reduce and eliminate from the library compounds, which did not present drug-like properties, by applying the strict “drug” filter. Then, the most probable tautomeric and protomeric state at pH 7.00 was attributed to each remaining compound through the “tautomers” and “fixpka” tools, respectively. Finally, three-dimensional coordinates were generated with CORINA Classic, and partial charges were attributed according to the MMFF94 [72] force field through the “molcharge” tool. 

### 4.7. Docking-Based Virtual Screening

The prepared compound library was docked onto the catalytic site of the I7L protease dimeric model using the Protein–Ligand ANT System (PLANTS) [73,74] program, which is free to use for academics, coupled with the ChemPLP [75] scoring function. The binding site was defined as a sphere of radius 12 Å centered around the center mass of Cys328, one of the three residues defining the catalytic triad. For each ligand, the best ten poses according to the scoring function were retained for further analysis. The same docking protocol was also applied to TTP-6171 [19], the only noncovalent I7L protease inhibitor available in the literature, which was used as a positive control.

Initially, docking poses were filtered based on the similarity of their interaction pattern with the top-scoring pose for the positive control TTP-6171. Specifically, the recently developed IFP_CS_ [43,76,77] scoring function was used to perform the comparison between the reference TTP-6171 pose and the query poses derived from the virtual screening. Each docking pose is encoded in a rx8 integer vector (where r is the number of protein residues and 8 is the number of possible protein–ligand interactions that are computed), and the cosine similarity between a reference pose and a query one is calculated. This results in a score that can range from −1 (indicating total congruence between the two binding patterns) and 0 (indicating total divergence).

Afterward, poses were filtered based on their electrostatic and van der Waals interaction energy, other than the percentage of the solvent-exposed surface. The first two elements were calculated through the related descriptors of MOE, while the third was computed through an SVL script provided by the MOE support team. Finally, the remaining poses were subjected to a round of visual inspection. All adopted filtering criteria, which were calibrated based on the reference docking pose for the TTP-6171, are summarized in Table 2. A heatmap reporting a per-residue decomposition of the electrostatic (kcal/mol) and hydrophobic (arbitrary units) contribution to the protein–ligand interaction energy for selected poses was generated by making use of an in-house SVL script.

### 4.8. MD-Based Post-Docking Refinement

To account for the protein flexibility, which is neglected in docking calculations, docking poses that passed all filters described in Section 4.5 were subjected to MD-based refinement. Specifically, each protein–ligand complex derived from the virtual screening and the reference docking pose for the TTP-6171 were prepared for MD simulations using the same protocol described in Section 4.2, both in the system setup and in the equilibration stage. Differently from the simulation of protein-only systems, ligand partial charges were attributed through the “antechamber” module using the AM1-BCC [78] method, ligand parameters were assigned according to the general amber force field (GAFF) [79] and harmonic positional restraints were added also to the ligand atoms in both equilibration stages. For the production stage, 3 independent, 3 ns MD replicates were carried out to relax the system in an explicit solvent environment. 

Generated trajectories were, then, analyzed by making use of an in-house Python script, calculating the ligand RMSF, the hydrophobic contribution to the interaction energy derived from the empirical Cyscore [80] scoring function, the number of hydrogen bonds calculated with VMD, and the interaction energy, calculated as the sum of the electrostatic and van der Waals terms, computed through the NAMD [81] Energy 1.4 plugin for VMD. 

## Figures and Tables

**Table 1:** Summary of principal descriptors extracted from the MD-based post-docking refinement of virtual screening derived poses for the 14 selected compounds, plus the reference TTP-6171 pose. The ligand RMSF, the average number of hydrogen bonds for each trajectory frame, the hydrophobic score, electrostatic and van der Waals contribution to the protein–ligand interaction energy, and the total interaction energy are reported. All descriptors are mediated across three independently run MD replicates.

Molecule	RMSF (Å)	Hydrogen Bonds (Average Per Frame)	Hydrophobic Score (kcal/mol)	Electrostatic Contribution (kcal/mol)	Van der Waals Contribution (kcal/mol)	Interaction Energy (ele + vdW) (kcal/mol)
TTP-6171	1.7	0.39	−1.27	−13.15	−43.15	−56.3
DB01748	1.11	0.33	−1.03	−24.07	−31.52	−55.59
DB02429	0.91	0.42	−1.11	−25.74	−32.58	−58.31
DB04888	1.7	0.62	−1.07	−35.46	−35.95	−71.42
DB07476	1	0.52	−0.58	−25.57	−31.4	−56.97
DB07531	2.54	0.15	−0.88	−15.51	−30.32	−45.83
DB07537	1.65	0.19	−1.41	−19.35	−36.64	−55.99
DB08399	1.2	1.18	−0.95	−19.91	−24.15	−44.06
DB08754	1.44	0.4	−1.22	−19.87	−33.97	−53.84
DB12027	1.39	0.44	−0.96	−13.85	−32.75	−46.6
DB12558	1.3	0.61	−1.36	−29.9	−41.37	−71.27
DB12882	1.54	0.88	−0.99	−66.94	−34.76	−101.69
DB13248	5.94	0.72	−0.12	−65.76	−12	−77.76
DB13948	1.66	0.65	−0.93	−29.17	−27.82	−56.99
DB16236	1.8	0.59	−0.6	−84.37	−31.02	−115.39

**Table 2:** Pose filtering criteria adopted for the docking-based virtual screening performed on the I7L protease dimeric model.

Observable	Criteria
IFP_CS_	≤−0.97
Electrostatic interaction energy	≤10 kcal/mol
Van der Waals interaction energy	≤10 kcal/mol
% ligand solvent exposure	≤15%
Visual inspection	Good shape complementarity with the pocket

## Data Availability

The I7L protease model structures reported and discussed in the article and the docking poses for TTP-6171 and the fourteen hit compounds derived from the virtual screening are available at github.com/molecularmodelingsection/MPOX_I7L_protease.

## Supplementary Materials

The following supporting information can be downloaded at: https://www.mdpi.com/article/10.3390/ijms24087119/s1.
