# Supplementary material for: Targeting the I7L Protease: A Rational Design for Anti-Monkeypox Drugs?
Source: Int J Mol Sci. 2023 Apr 12;24(8):7119. doi: 10.3390/ijms24087119 (PMC10138331; doi:10.3390/ijms24087119)
Supplement: Supplementary file 1 [file ijms-24-07119-s001.zip › ijms-2283540-supplementary/ijms-2283540-supplementary.pdf]

Supplementary Information

Targeting the I7L protease: towards the rational design of anti-Monkeypox drugs?

Andrea Dodaro , Matteo Pavan and Stefano Moro

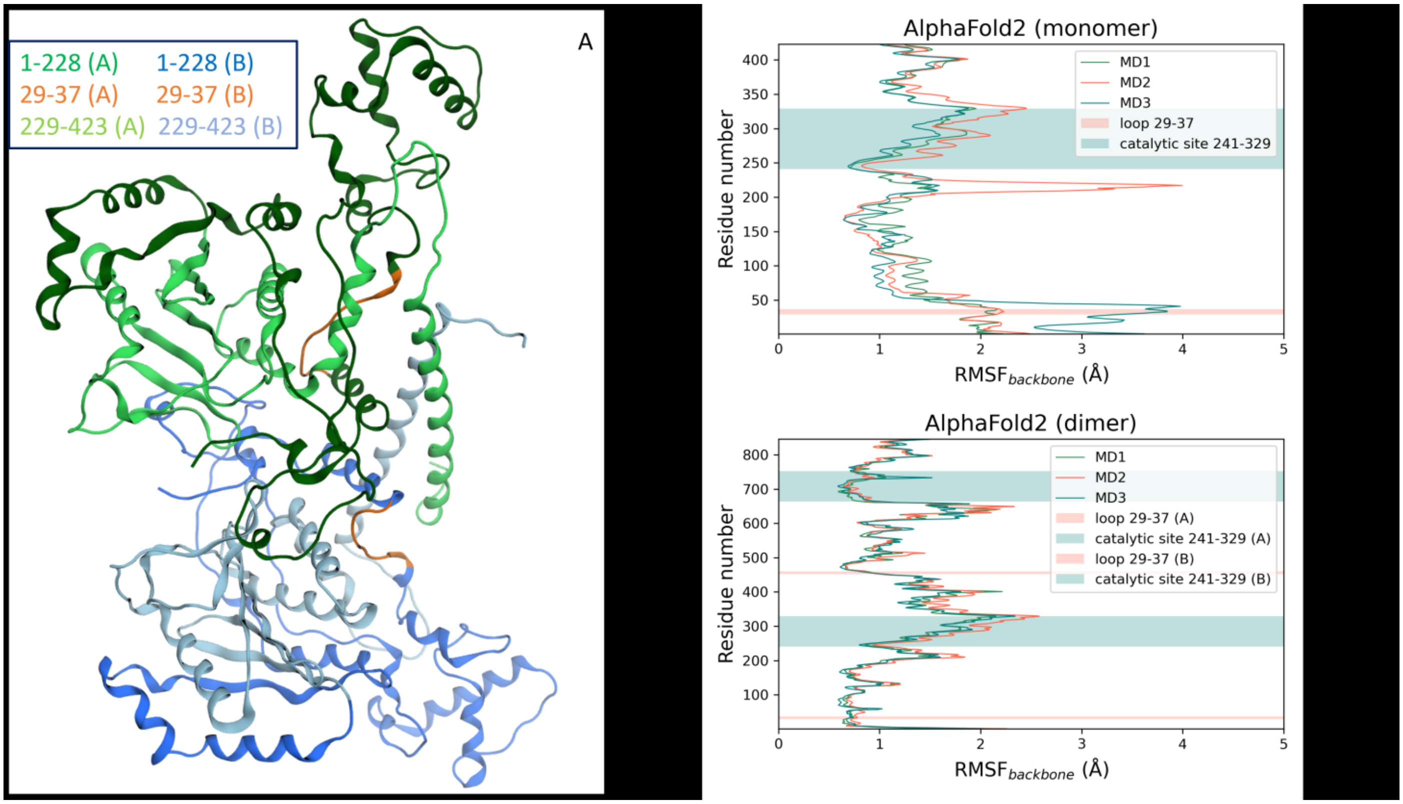

Figure S1. a) structure of the AlphaFold2 model of the I7L protease: the protein is represented in ribbon, color-coded to the corresponding domain/motif; b) per-residue RMSF profile for the monomeric AlphaFold2 model; c) per-residue RMSF profile for the dimeric AlphaFold2 model.

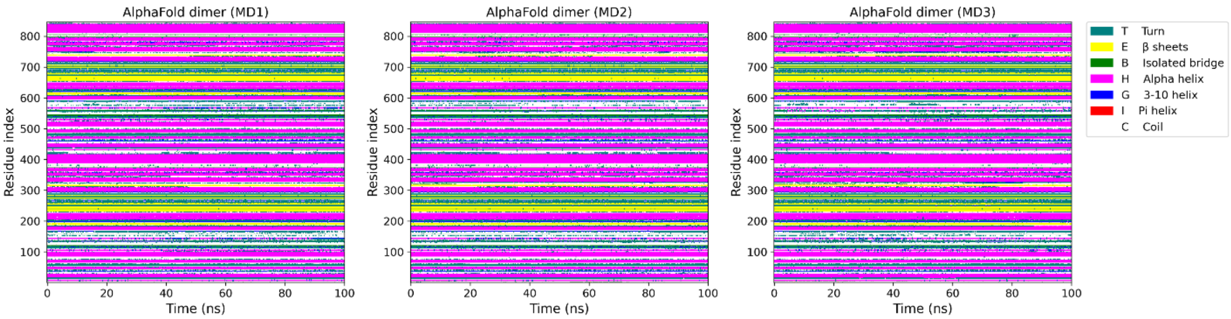

Figure S2. time-dependent evolution of the secondary structure for the dimeric AlphaFold2 model of the I7L protease, computed through the appropriate VMD module, for each classic MD simulation.

Table S1. Results for the in-silico mutagenesis experiment conducted through the "alanine scanning" module of the Molecular Operating Environment (MOE) 2022.02 suite. For each investigated mutant, reported in the original publication by Byrd et al., the variation in the protein potential energy (dStability = Epot MUT – Epot WT) is reported.

Mutation	H241A	W242A	D248A	D258A	Q322A	C328A	G329A
dStability (kcal/mol)	1.37	2.06	1.09	0.55	1.22	0.74	0.58

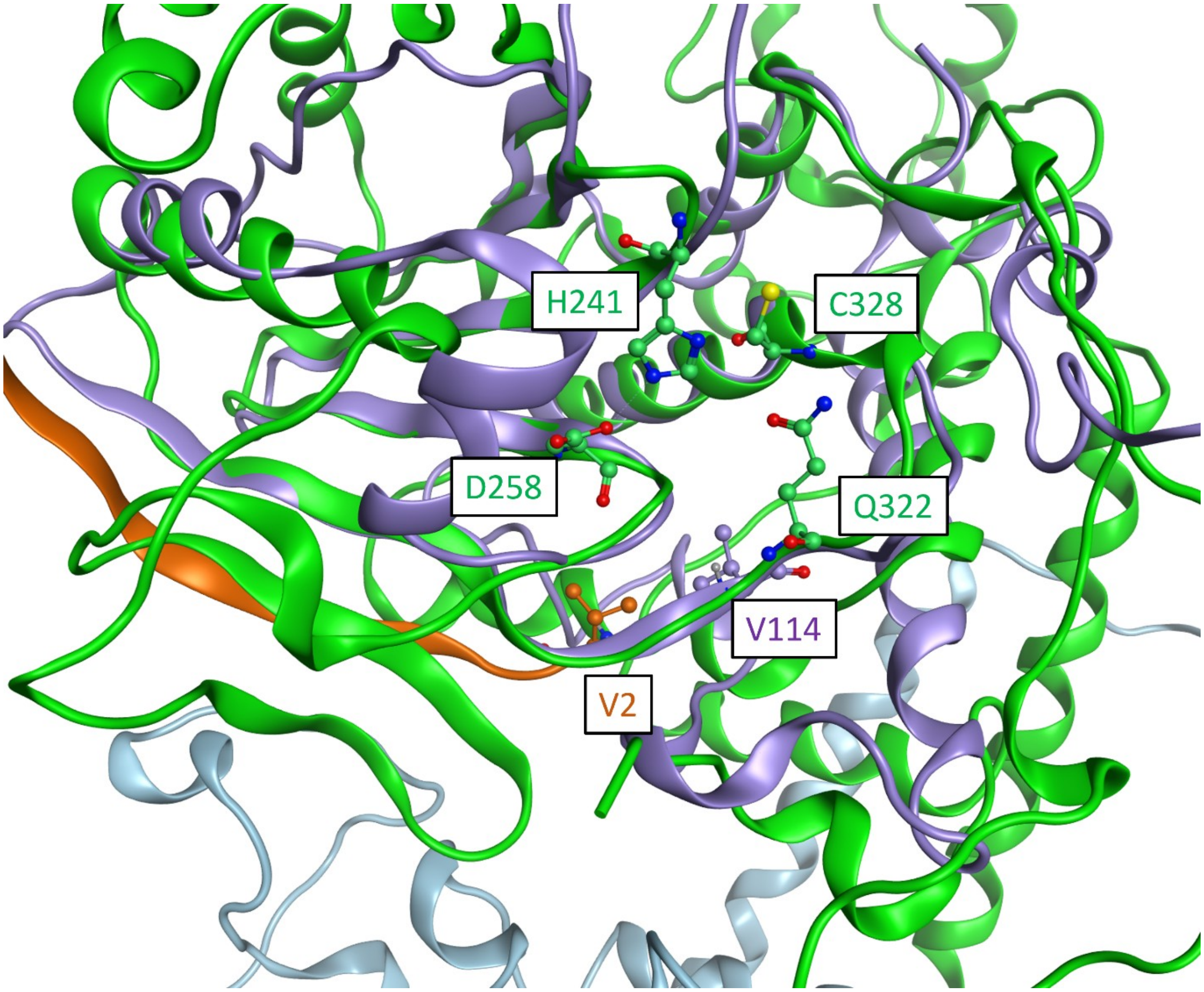

Figure S3. superposition between the structure of the human adenovirus 2 protease (violet, PDB ID: 1AVP) in complex with peptide cofactor pVIc (orange) superposed to AlphaFold2's dimeric model of the I7L protease (green, chain A; light blue, chain B). For reference, the catalytic triad and the glutamine residue forming the oxyanion hole, are shown in ball and stick. The conserved valine residue (V114) in the human adenovirus protease 2 and the valine residue necessary for the allosteric activation of the protease on the pVIc cofactor (V2) are also highlighted.

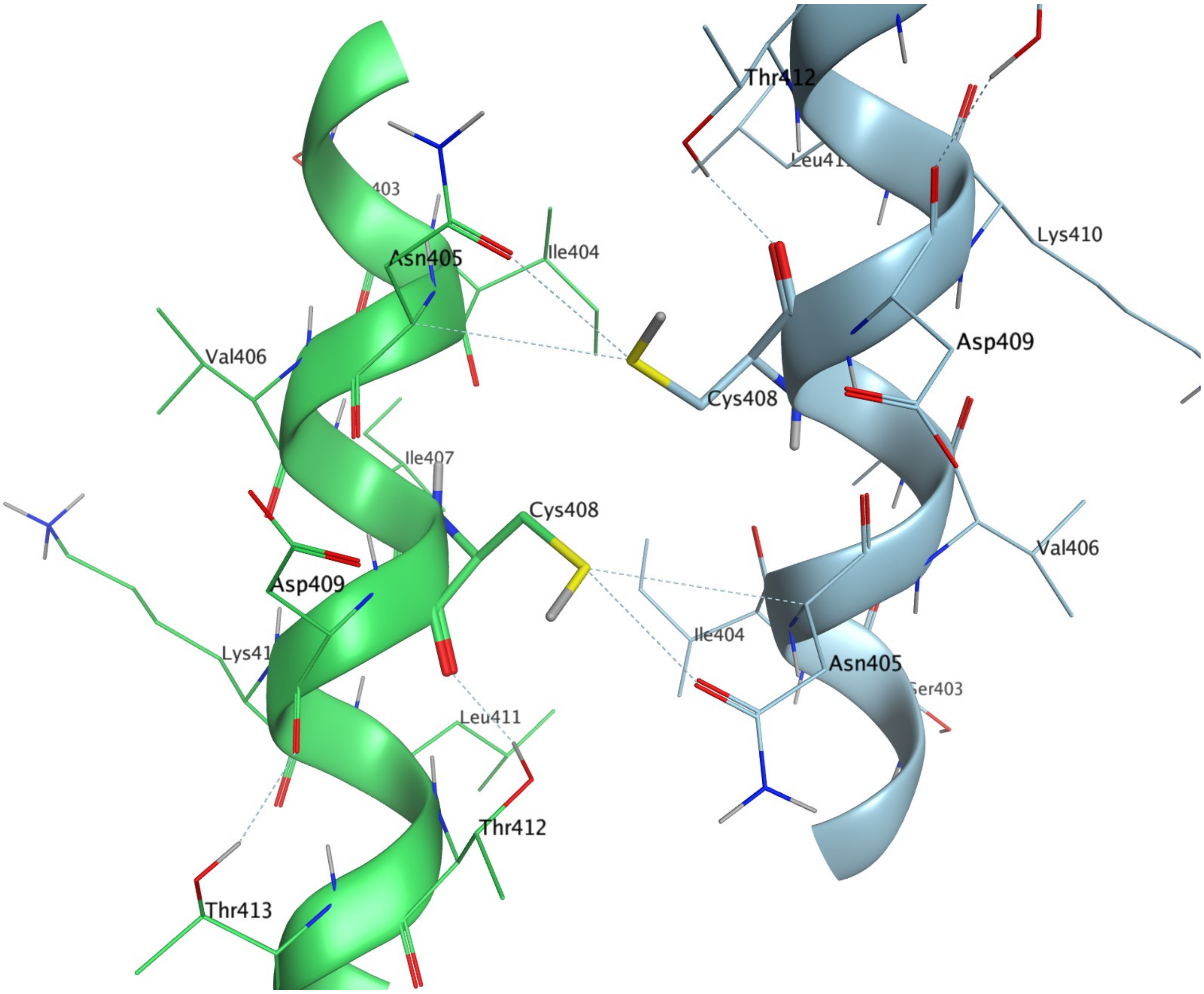

Figure S4. detail of the interface region between the two C-terminal alpha helices in proximity of residue C408: chain A is colored in green, while chain B is colored in light blue.

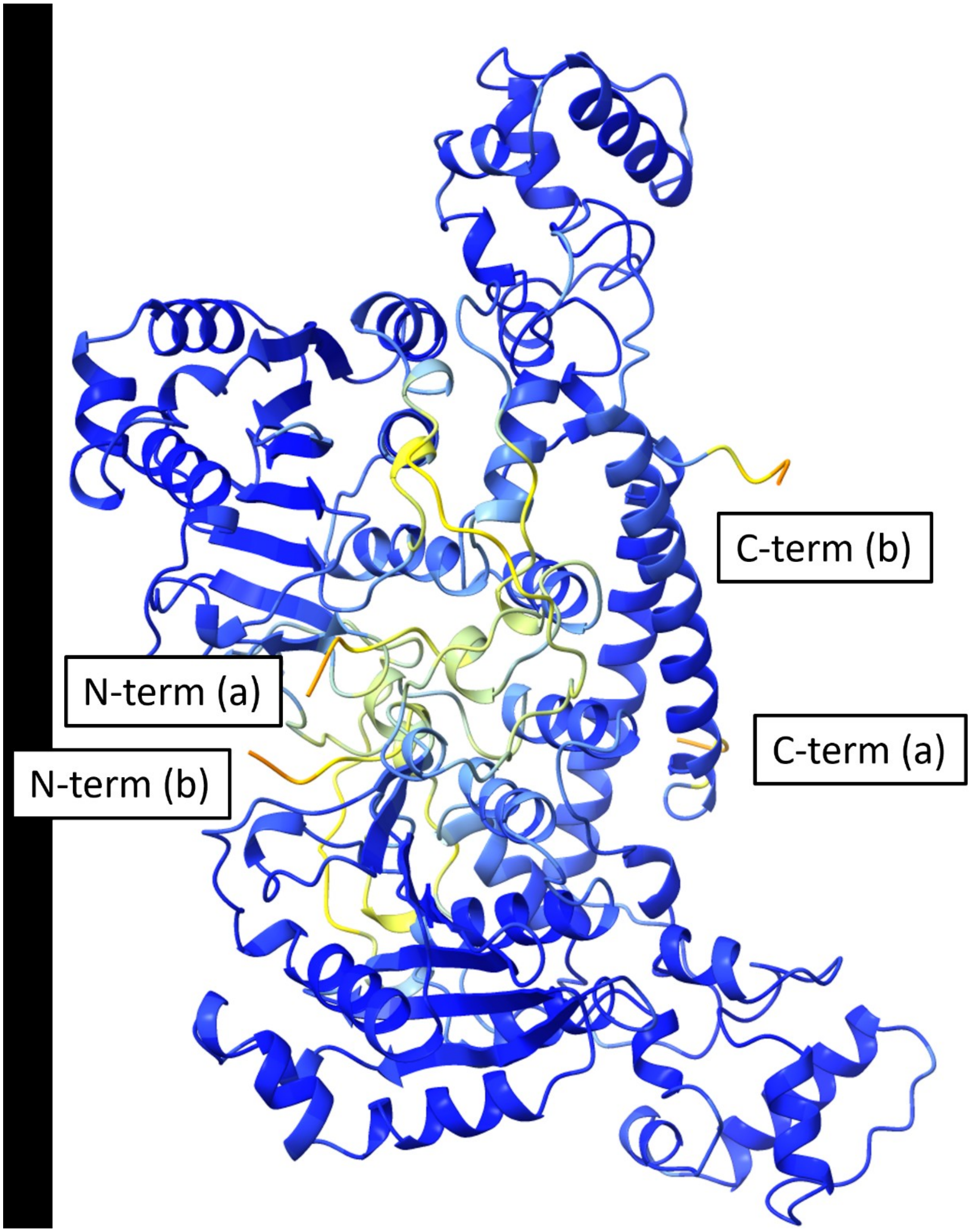

Figure S5. AlphaFold2 dimeric model of the I7L protease color-mapped according to the pLDDT score: regions associated with high confidence in the structural prediction (pLDDT > 90) are colored in blue, regions associated with good confidence in the prediction (90 > pLDDT > 70) are colored in light blue, regions associated with low confidence in the prediction (70 > pLDDT > 50) are colored in yellow, while regions where the prediction is not reliable (pLDDT < 50) are colored in shades of red.

Table S2. Report about the distances between the sulphur atoms of the C408 sidechains sampled through the MD simulations of the AlphaFold2 dimeric model of the I7L protease.

	MD1	MD2	MD3
Min distance (Å)	3.51	3.40	3.40
Mean distance (Å)	4.10	4.14	4.12
Max distance (Å)	5.00	5.28	5.13

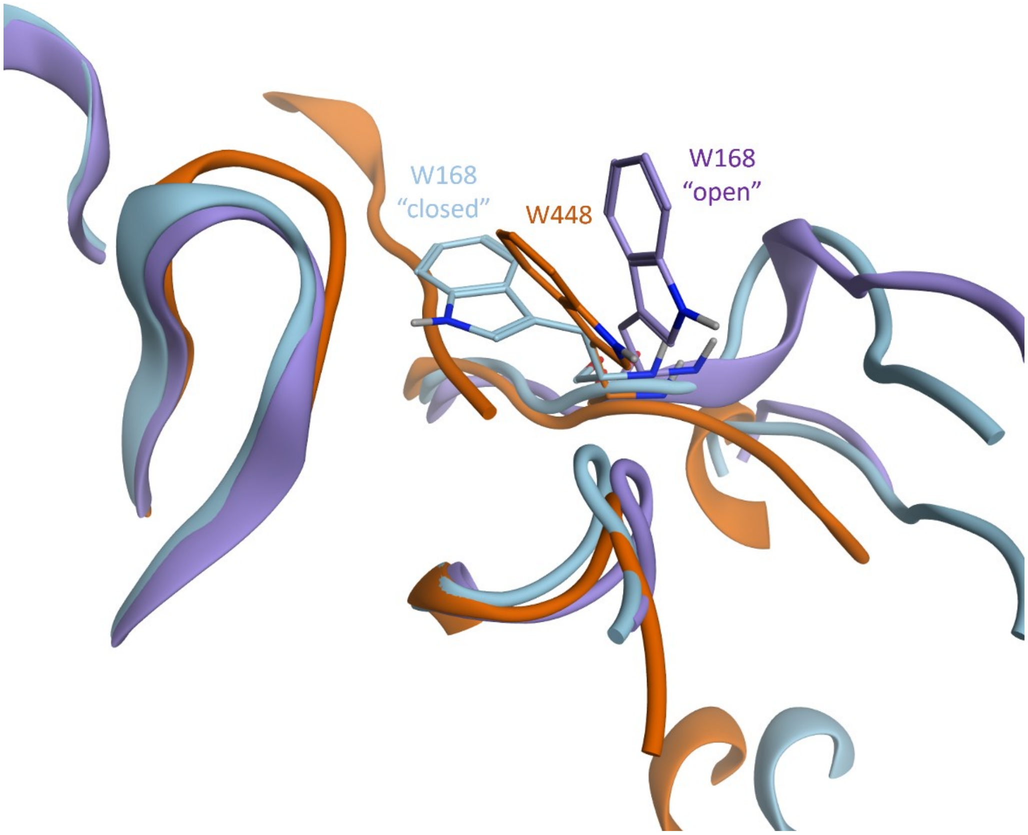

Figure S6. superposition between the "closed" conformation of Trp168 (light blue), the "open" conformation of Trp168 (violet), and the complex between the s. cerevisiae Ulp-1 protease and its substrate (orange, PDB ID: 1EUV). For reference, Trp448 from the Ulp-1 protease is also shown.

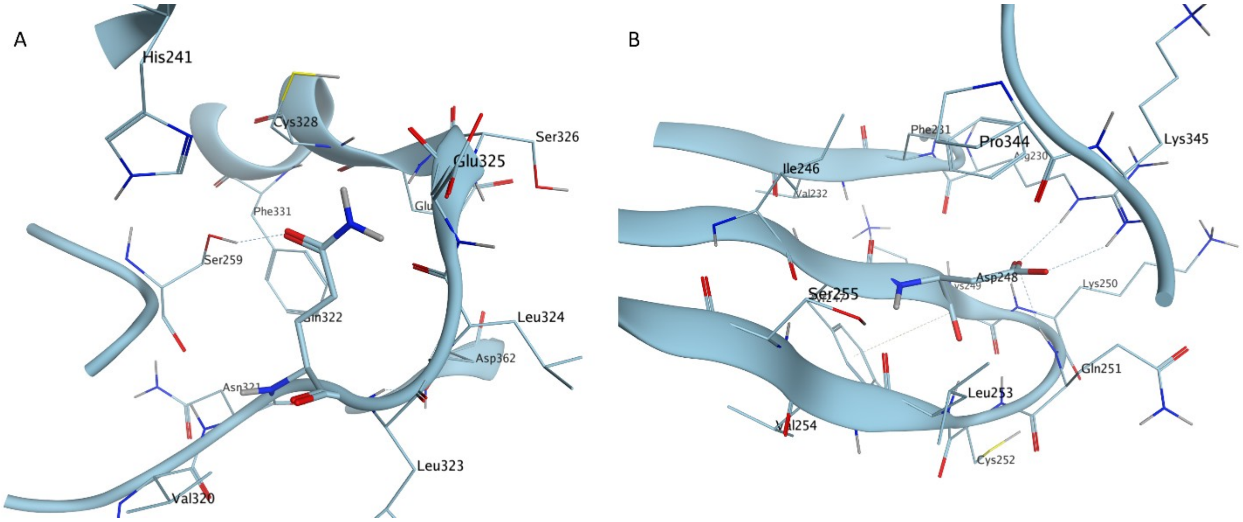

Figure S7. a) detail of the surroundings of G322 in the AlphaFold2's dimeric model of the I7L protease; b) detail of the surroundings of D248 in the AlphaFold2's dimeric model of the I7L protease.

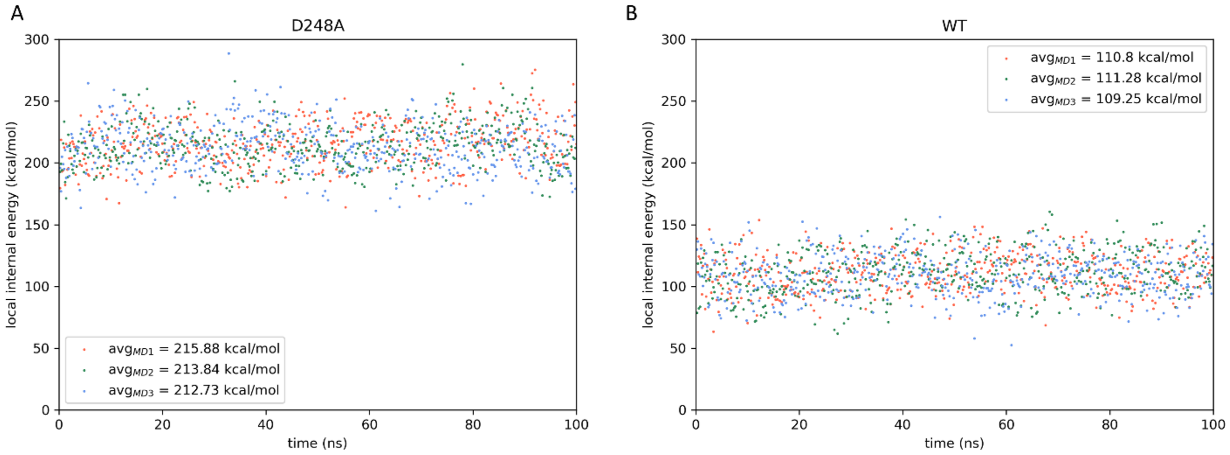

Figure S8. internal energy analysis computed on MD simulations for the AlphaFold2's dimeric model of the I7L protease. The internal energy is calculated for residues within 5 Å of the aminoacid involved in the mutagenesis study (D248) and is carried out through the NAMD Energy plugin for VMD according to the ff14SB force field. a) energetic profile for the D248A mutant. B) energetic profile for the wild-type protein.

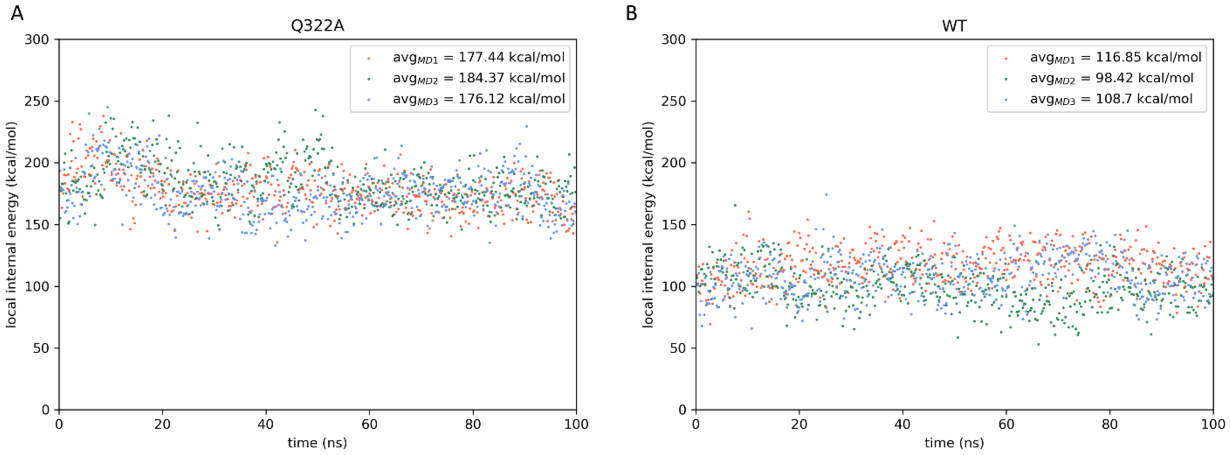

Figure S9. internal energy analysis computed on MD simulations for the AlphaFold2's dimeric model of the I7L protease. The internal energy is calculated for residues within 5 Å of the aminoacid involved in the mutagenesis study (Q322) and is carried out through the NAMD Energy plugin for VMD according to the ff14SB force field. a) energetic profile for the Q322A mutant. B) energetic profile for the wild-type protein.

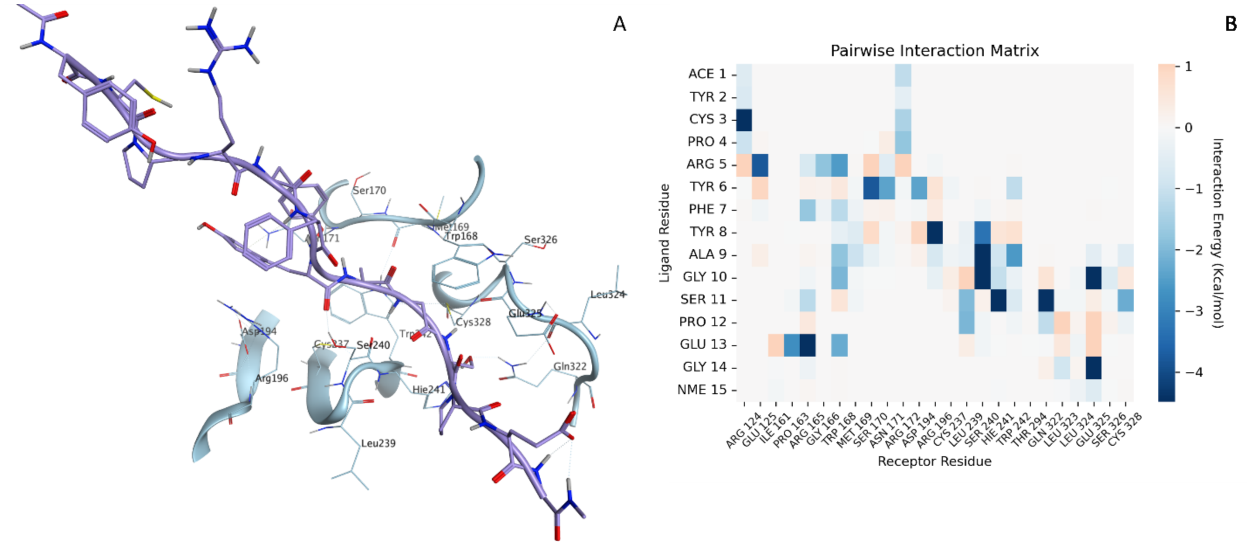

Figure S10. modeling of the interaction between substrate peptide P4a (G614-S615) and the catalytic site of the I7L protease. a) ligand pose extracted from the last frame of the representative trajectory (MD3), i.e., the one with the receptor-ligand interaction energy calculated through the MMGBSA method nearest to the average value across three independent replicates. b) pairwise interaction energy matrix: receptor residues are reported on the horizontal axis, while ligand residues are reported on the vertical axis.

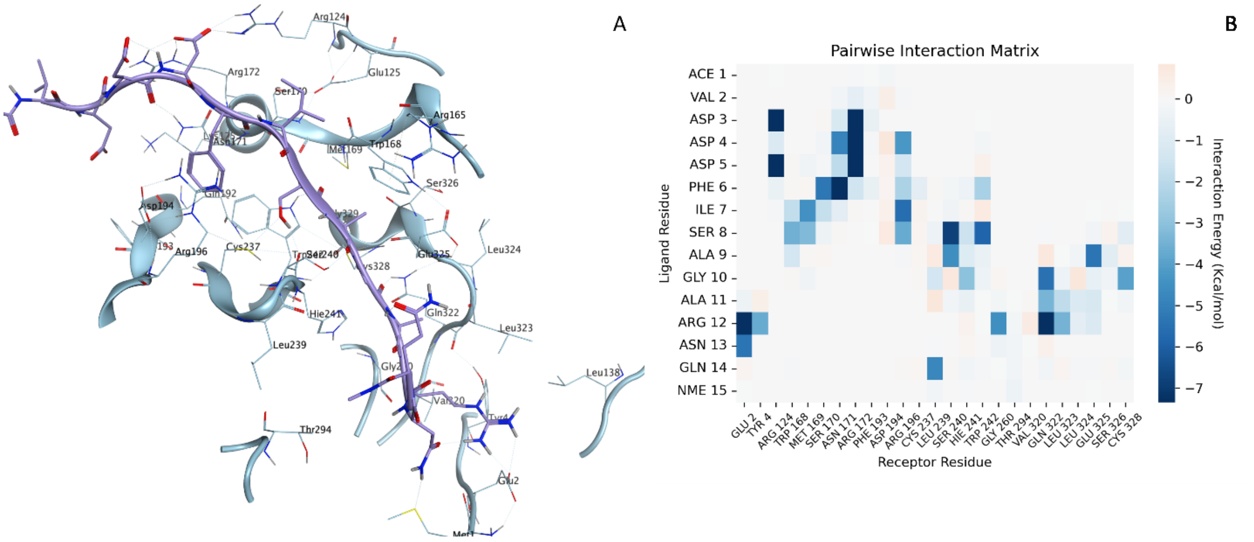

Figure S11. modeling of the interaction between substrate peptide P4b (G61-A62) and the catalytic site of the I7L protease. a) ligand pose extracted from the last frame of the representative trajectory (MD2), i.e., the one with the receptor-ligand interaction energy calculated through the MMGBSA method nearest to the average value across three independent replicates. b) pairwise interaction energy matrix: receptor residues are reported on the horizontal axis, while ligand residues are reported on the vertical axis.

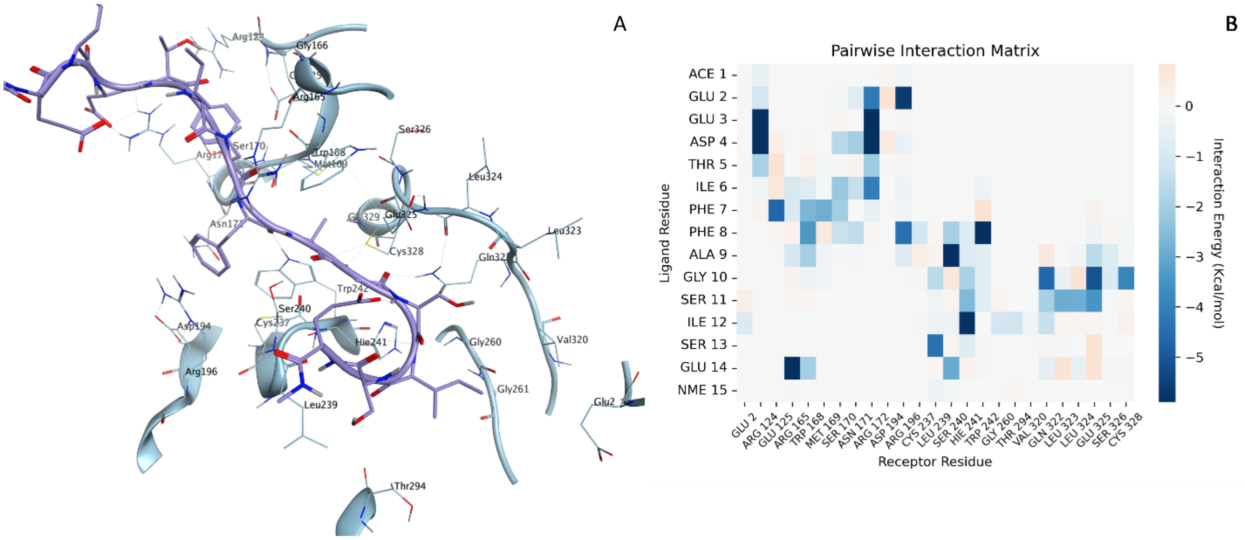

Figure S12. modeling of the interaction between substrate peptide VP8 (G18-S19) and the catalytic site of the I7L protease. a) ligand pose extracted from the last frame of the representative trajectory (MD2), i.e., the one with the receptor-ligand interaction energy calculated through the MMGBSA method nearest to the average value across three independent replicates. b) pairwise interaction energy matrix: receptor residues are reported on the horizontal axis, while ligand residues are reported on the vertical axis.

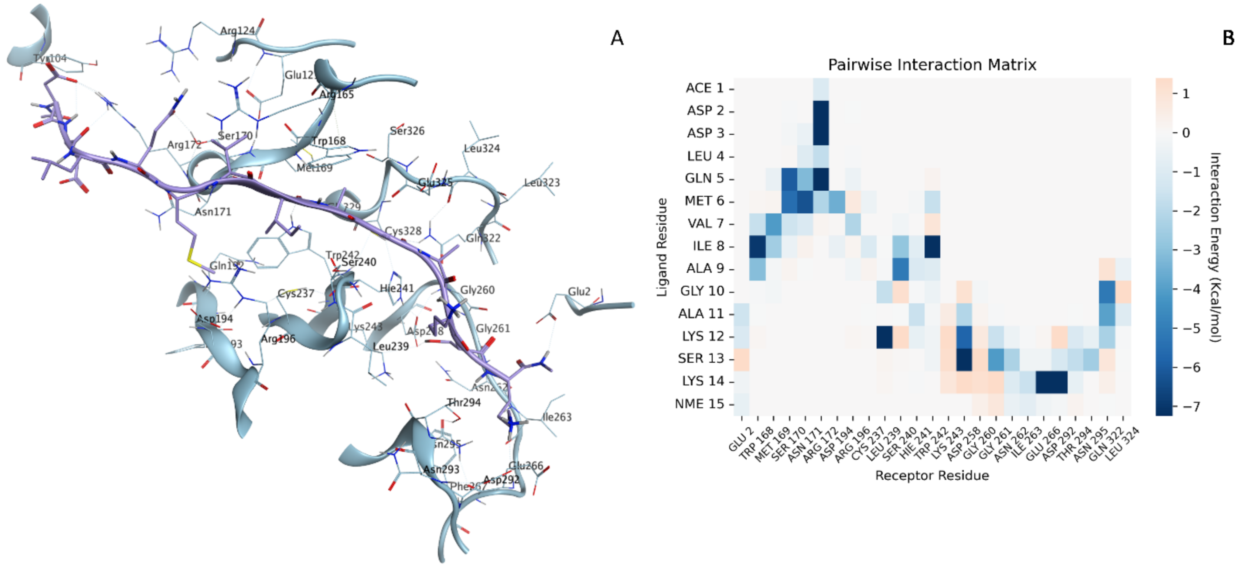

Figure S13. modeling of the interaction between substrate peptide VP8 (G32-S33) and the catalytic site of the I7L protease. a) ligand pose extracted from the last frame of the representative trajectory (MD3), i.e., the one with the receptor-ligand interaction energy calculated through the MMGBSA method nearest to the average value across three independent replicates. b) pairwise interaction energy matrix: receptor residues are reported on the horizontal axis, while ligand residues are reported on the vertical axis.

Table S3. Receptor-ligand interaction energy (kcal/mol) calculated through the MMGBSA method for each substrate peptide sequence.

	P4a	P4b	VP8 (G18-S19)	VP8 (G32-A33)
MD1	-44.28	-67.45	-68.91	-80.77
MD2	-37.67	-71.48	-65.57	-71.85
MD3	-39.91	-83.69	-64.51	-74.65

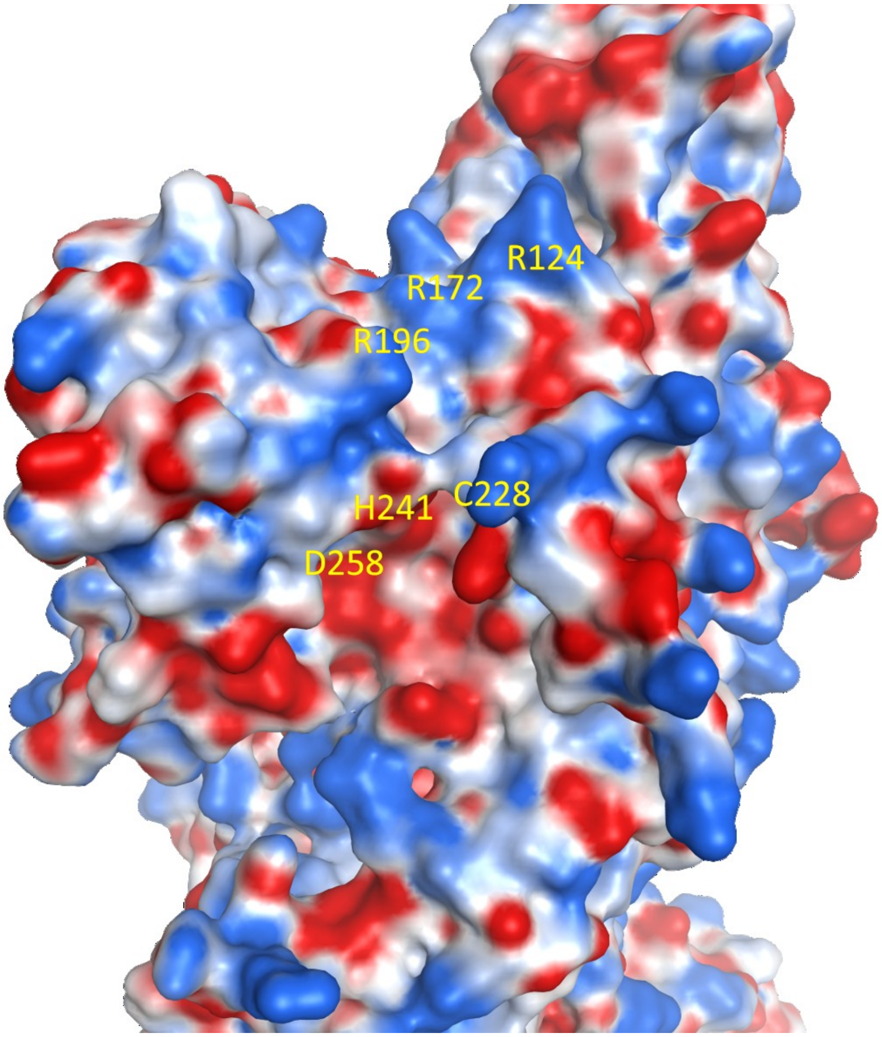

Figure S14. projection of the coulombic electrostatic potential on the Connolly surface of the dimer I7L protease model generated by AlphaFold2. The positions of the catalytic triad (C228, H241, and D258) and the electropositive exosite 1 (R124, R172, and R196) are highlighted.

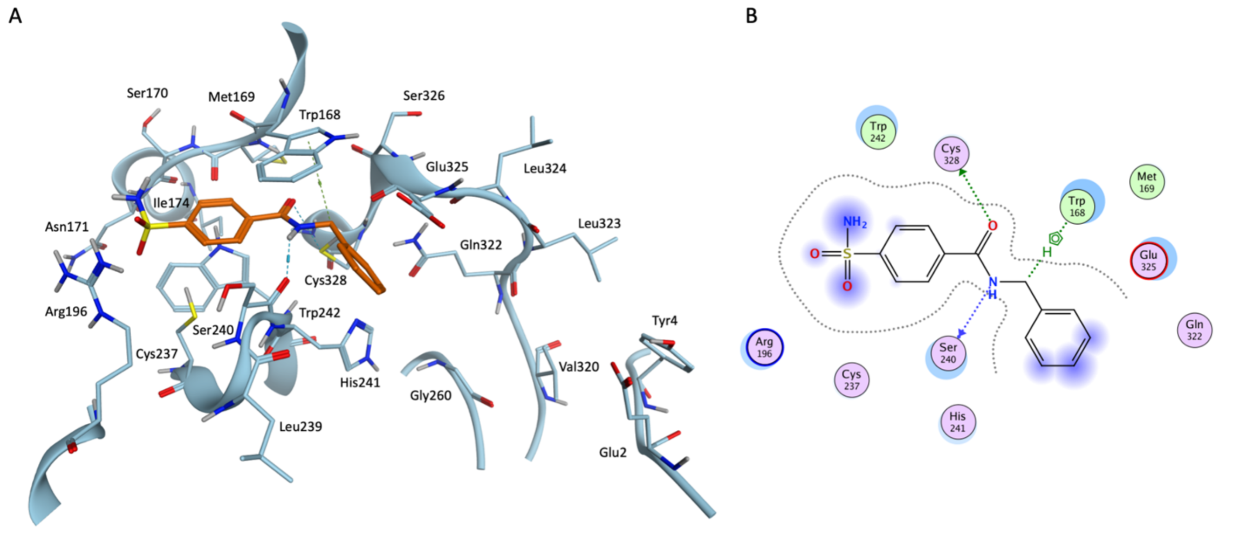

Figure S15. a) three-dimensional representation of the docking pose for compounds DB01748. b) bidimensional representation of the docking pose for compounds DB01748.

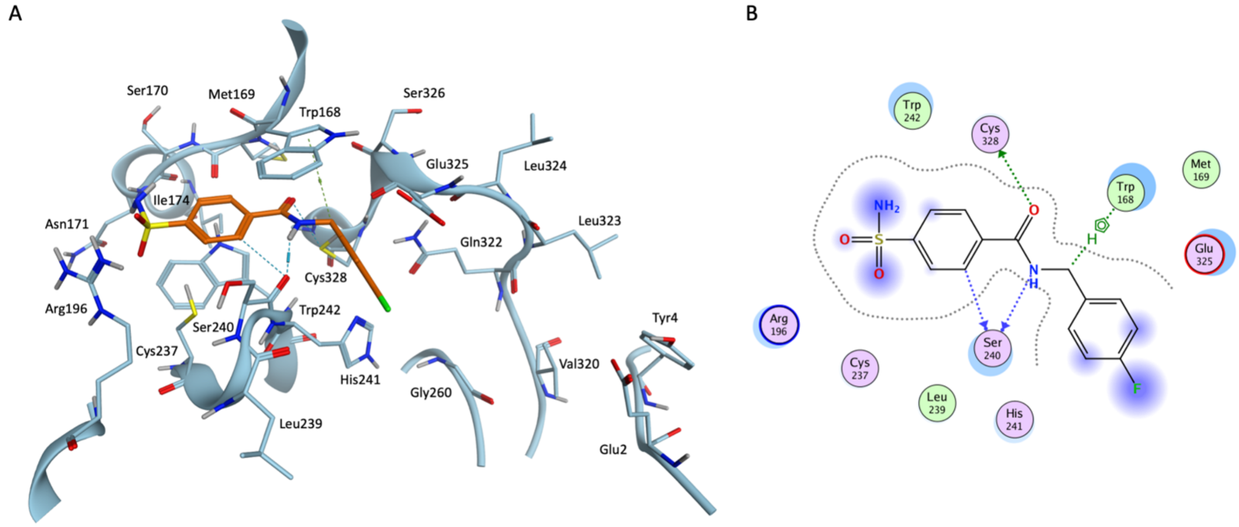

Figure S16. a) three-dimensional representation of the docking pose for compounds DB02429. b) bidimensional representation of the docking pose for compounds DB02429.

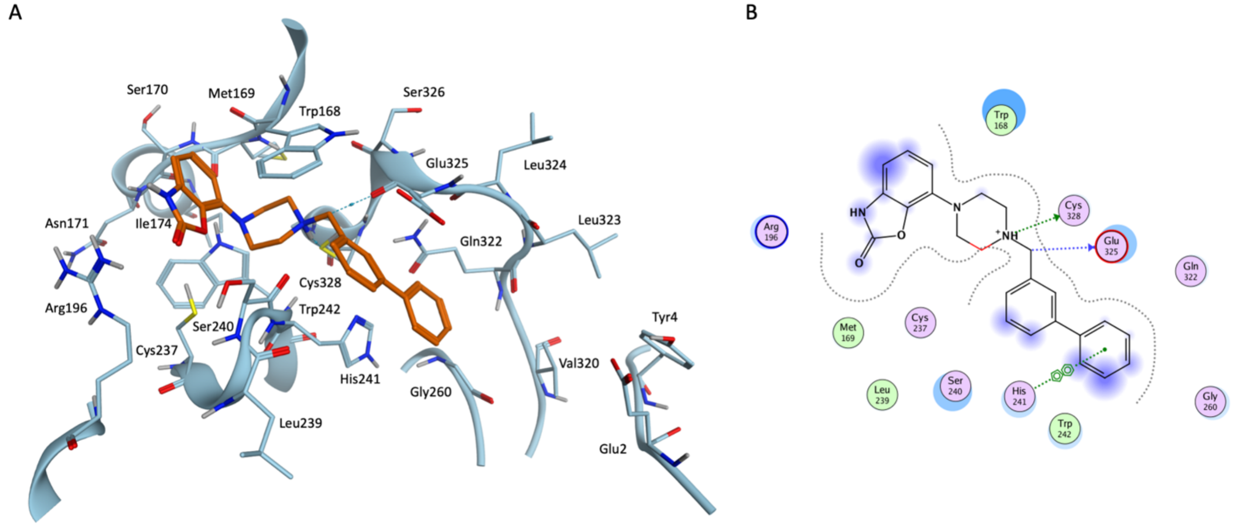

Figure S17. a) three-dimensional representation of the docking pose for compounds DB04888. b) bidimensional representation of the docking pose for compounds DB04888.

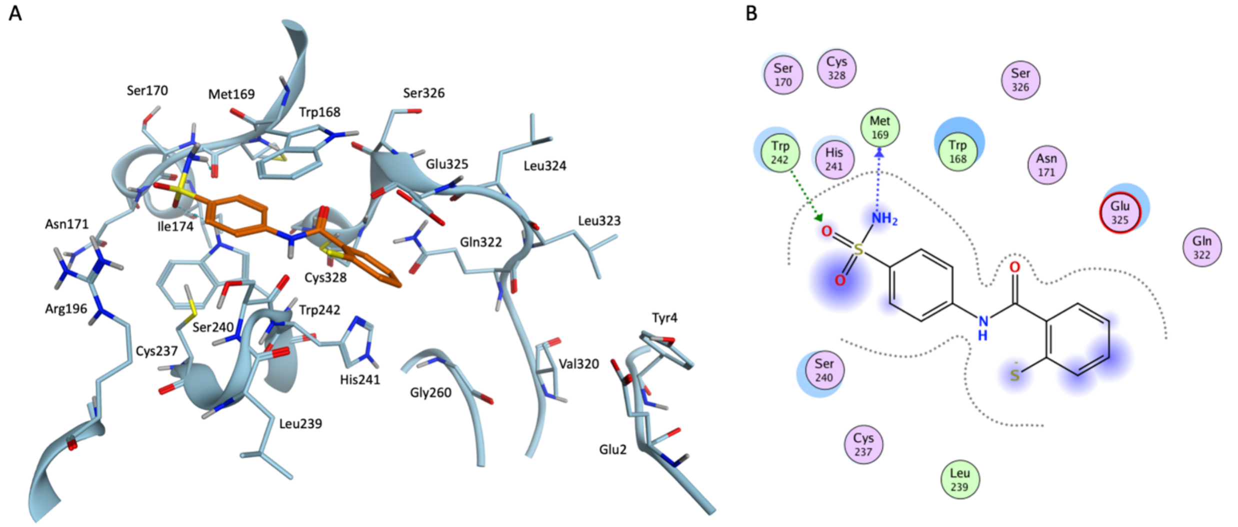

Figure S18. a) three-dimensional representation of the docking pose for compounds DB07476. b) bidimensional representation of the docking pose for compounds DB07476.

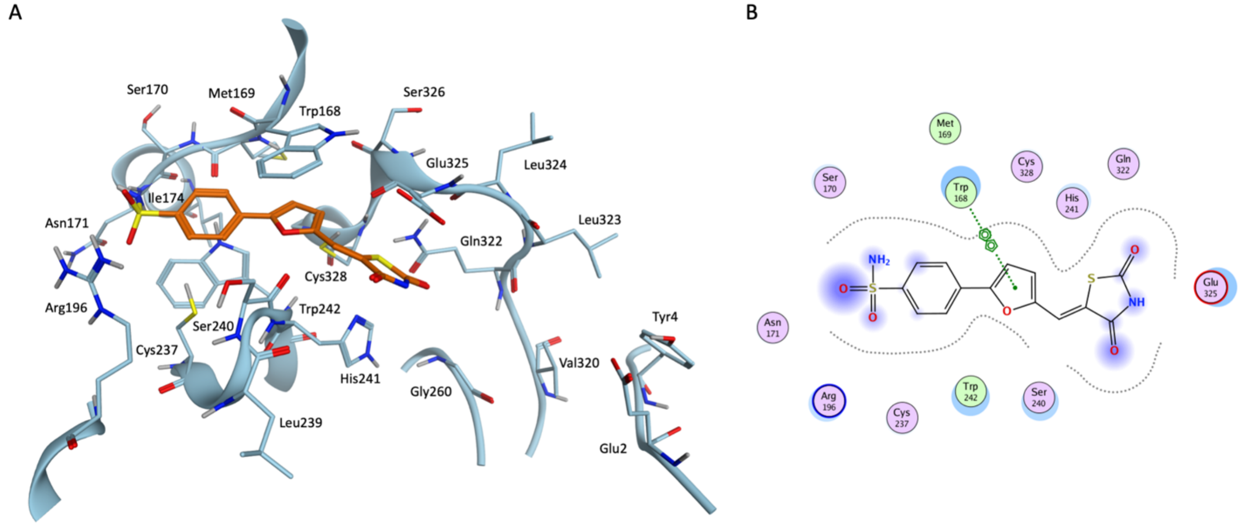

Figure S19. a) three-dimensional representation of the docking pose for compounds DB07531. b) bidimensional representation of the docking pose for compounds DB07531.

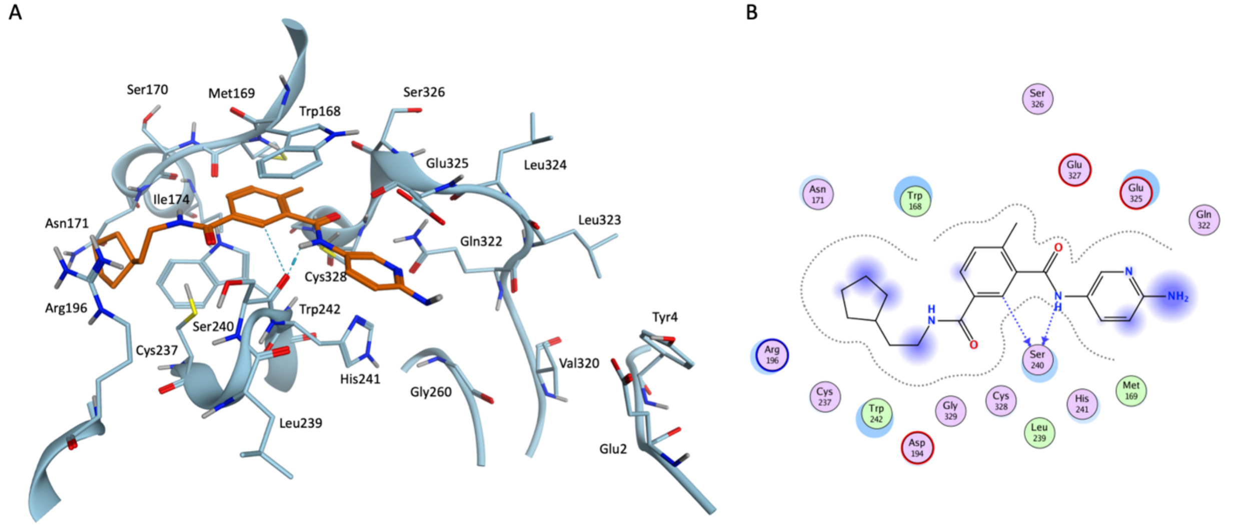

Figure S20. a) three-dimensional representation of the docking pose for compounds DB07537. b) bidimensional representation of the docking pose for compounds DB07537.

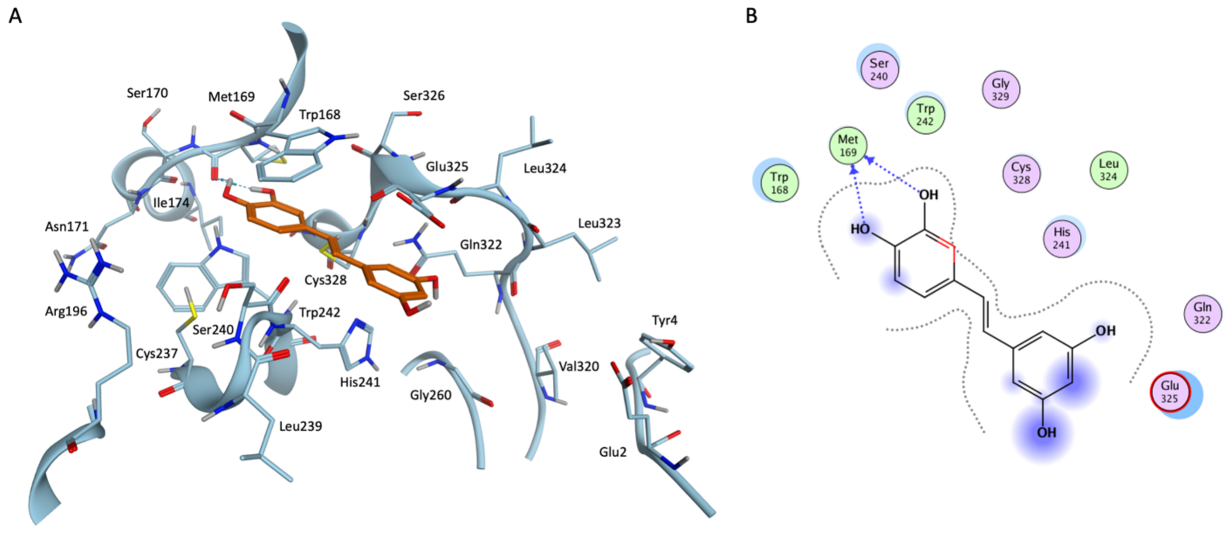

Figure S21. a) three-dimensional representation of the docking pose for compounds DB08399. b) bidimensional representation of the docking pose for compounds DB08399.

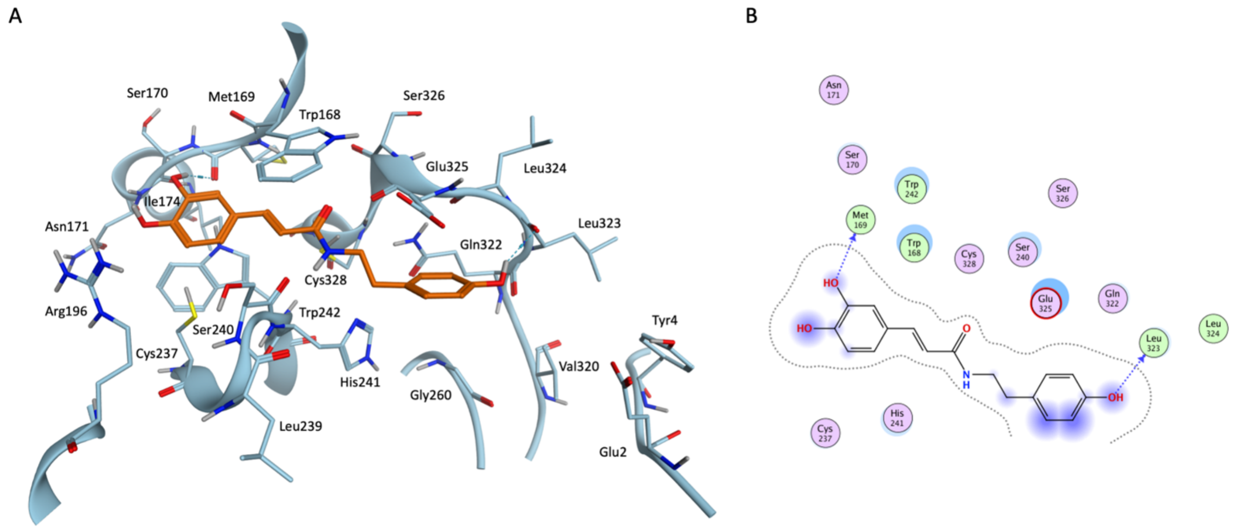

Figure S22. a) three-dimensional representation of the docking pose for compounds DB08754. b) bidimensional representation of the docking pose for compounds DB08754.

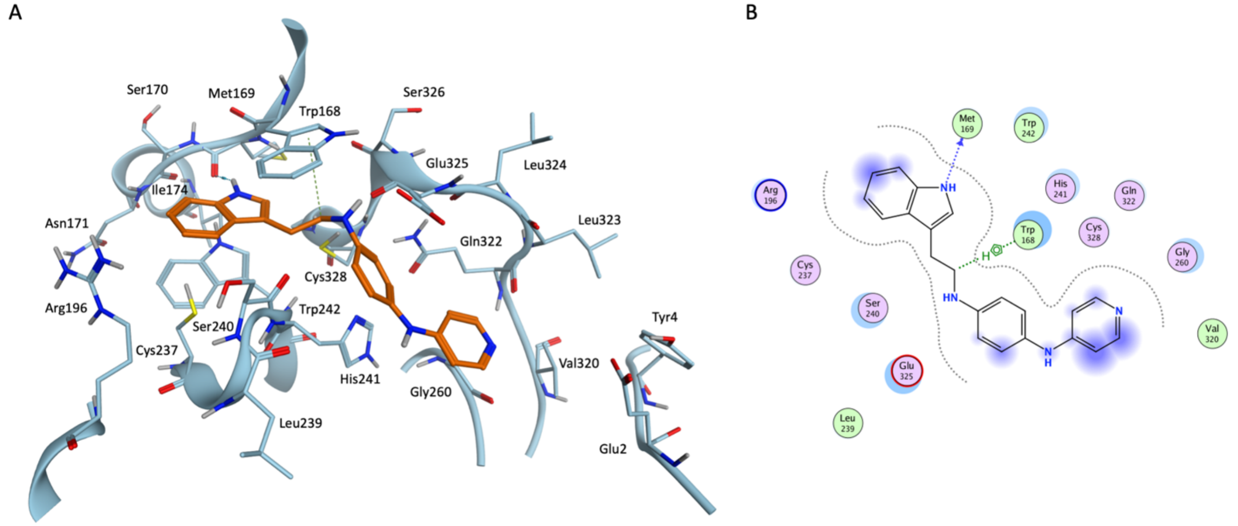

Figure S23. a) three-dimensional representation of the docking pose for compounds DB12027. b) bidimensional representation of the docking pose for compounds DB12027.

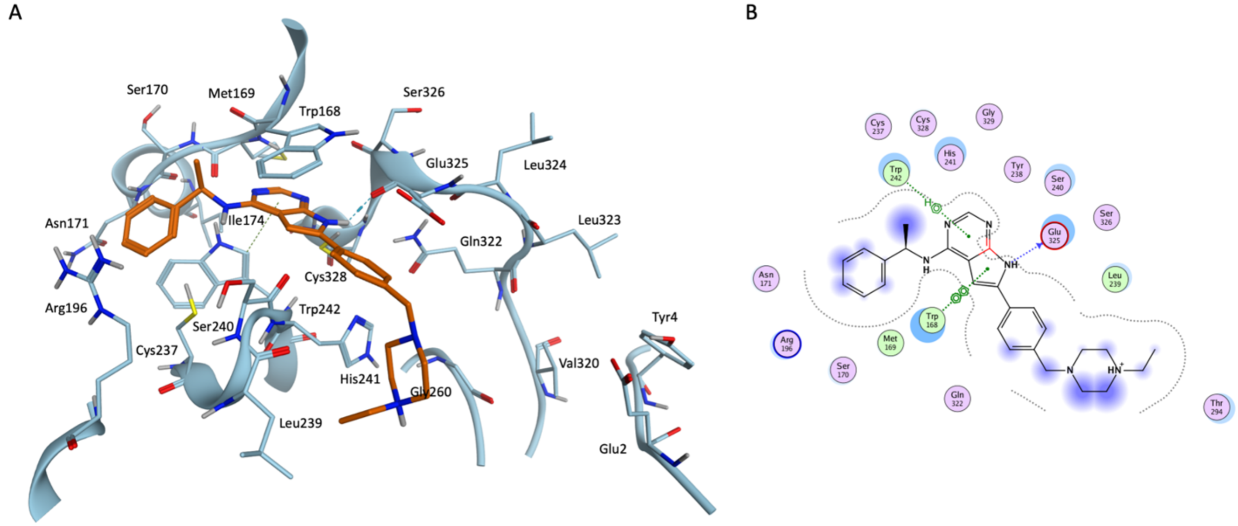

Figure S24. a) three-dimensional representation of the docking pose for compounds DB12558. b) bidimensional representation of the docking pose for compounds DB12558.

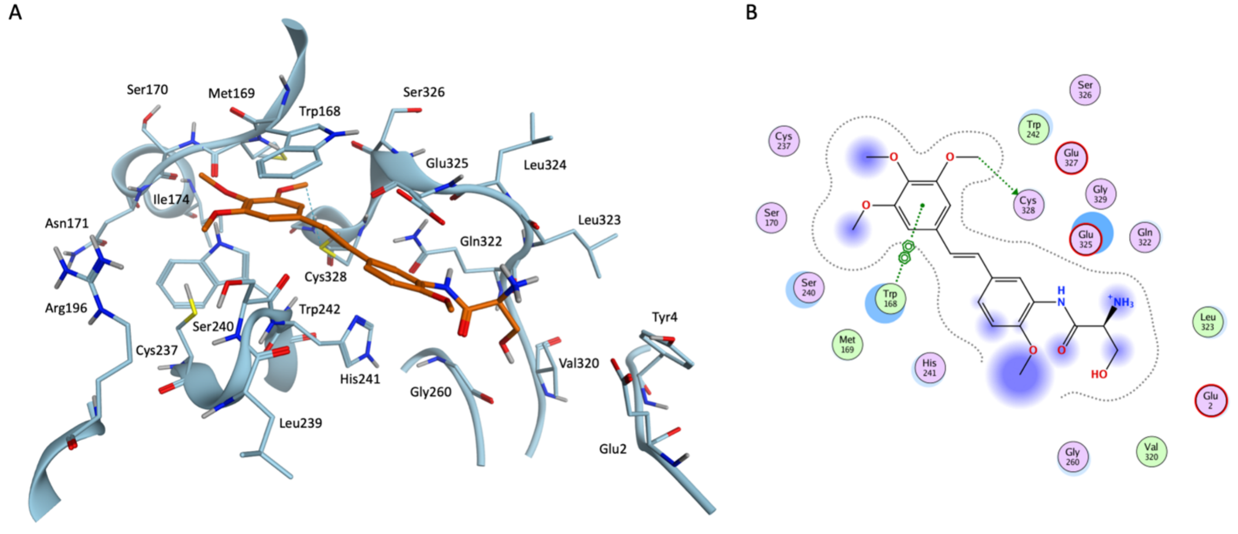

Figure S25. a) three-dimensional representation of the docking pose for compounds DB12882. b) bidimensional representation of the docking pose for compounds DB12882.

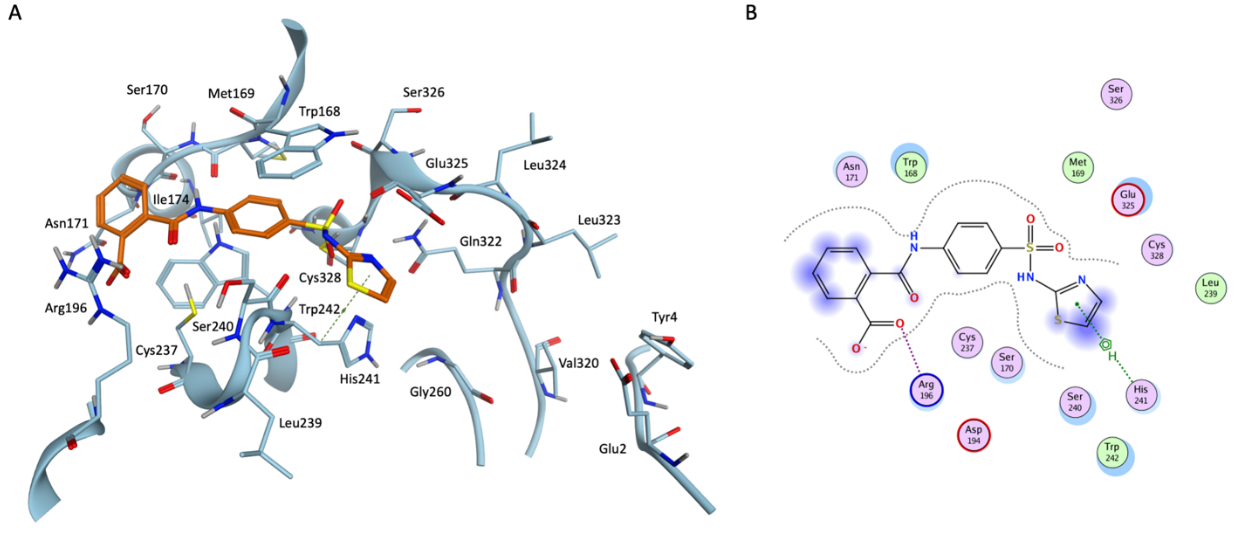

Figure S26. a) three-dimensional representation of the docking pose for compounds DB13248. b) bidimensional representation of the docking pose for compounds DB13248.

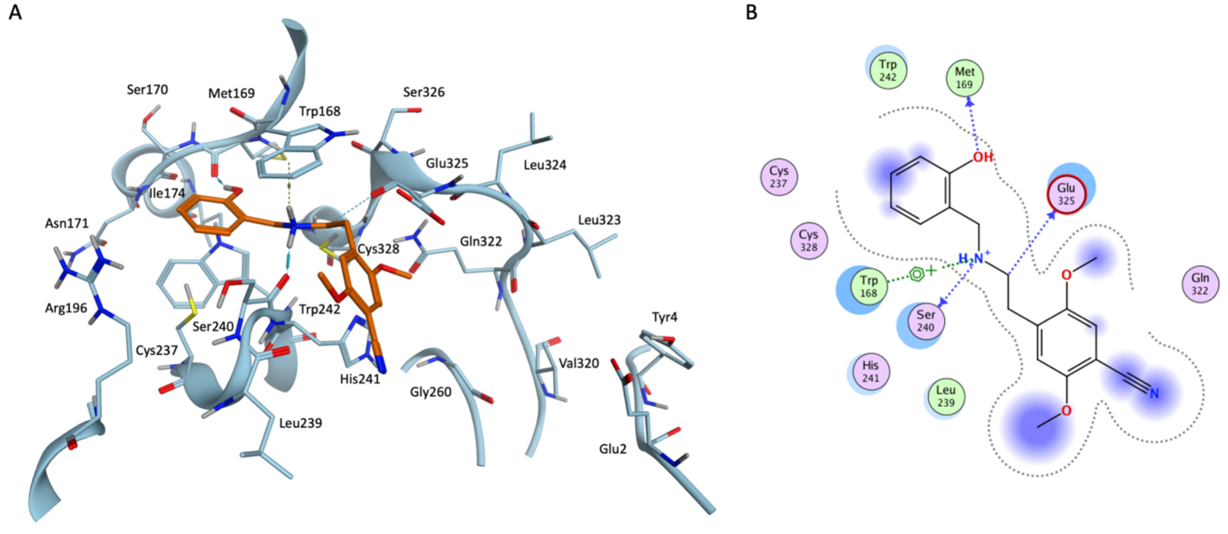

Figure S27. a) three-dimensional representation of the docking pose for compounds DB13948. b) bidimensional representation of the docking pose for compounds DB13948.

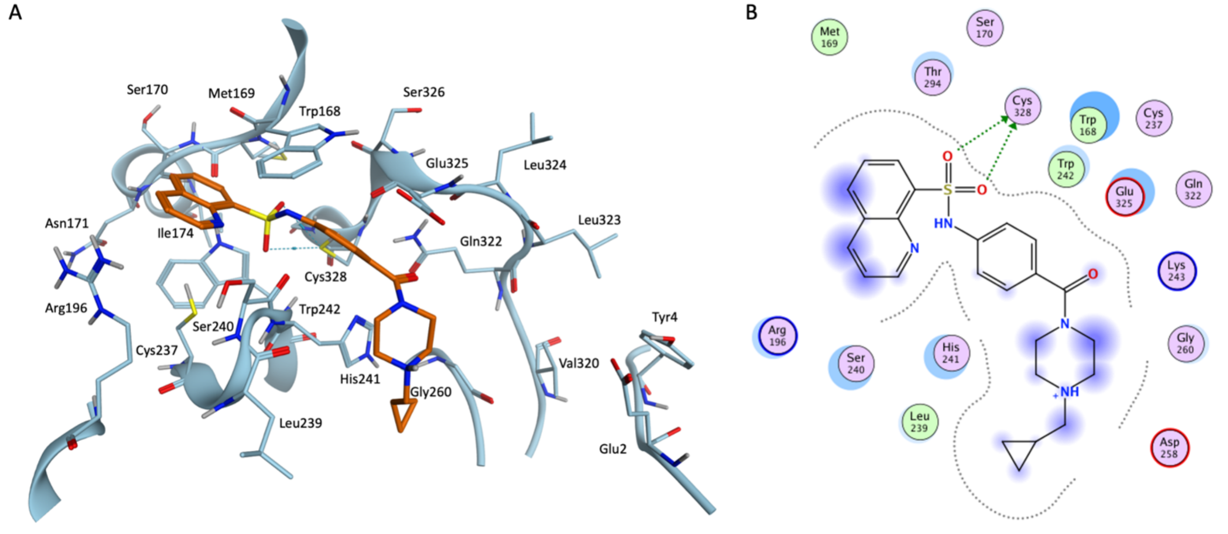

Figure S28. a) three-dimensional representation of the docking pose for compounds DB16236. b) bidimensional representation of the docking pose for compounds DB16236.

Table S4. Relevant information for the 14 compounds selected through the docking-based virtual screening carried out on the DrugBank database against the catalytic site of the monkeypox virus I7L protease. Compound names are color-coded based on the library subset to which they belong: approved drugs are highlighted in green, investigational compounds are highlighted in yellow, while experimental compounds are highlighted in red.

PDB code	Compound name	Mechanism of action	PDB code	Structure
DB01748		Carbonic anhydrase inhibitor	1G4O	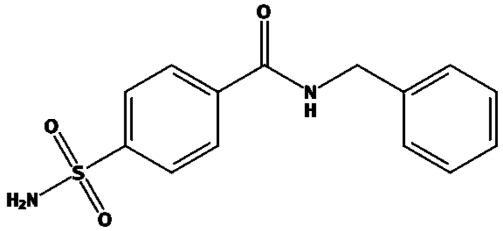
DB02429		Carbonic anhydrase inhibitor	1I9L	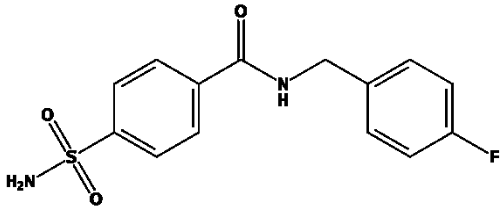
DB04888	Bifeprunox	5-HT1 agonist with partial agonism against to dopamine D2 receptors		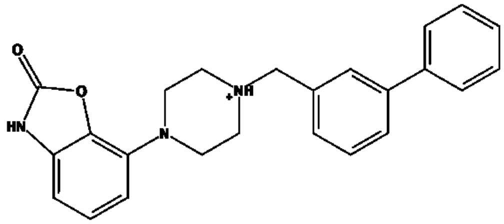
DB07476		Carbonic anhydrase inhibitor	2HD6	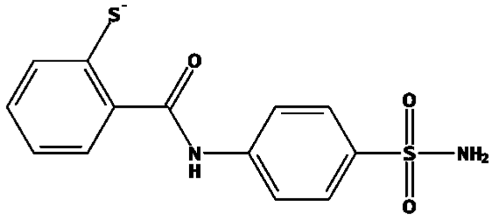

DB07531		cdk2 inhibitor	2UZO	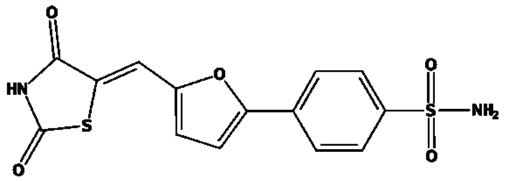
DB07537		Tyrosine kinase inhibitor (c-Kit)	3CPB	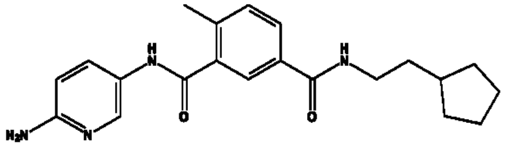
DB08399	Piceatannol	Natural resveratrol analog	2JJ1<br>4HD8<br>5U97<br>7CCV	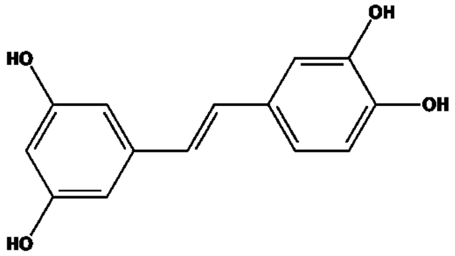
DB08754	N-Caffeoyl O-methyltyramine	Peptide deformylase inhibitor	2EW6	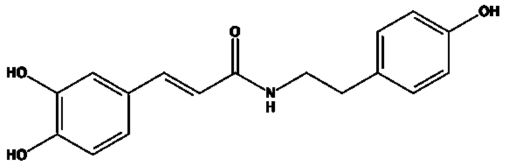
DB12027	Serdemetan<br>(JNJ-26854165)	Prevents the HD2-P53 interaction, inhibiting the degradation of p53		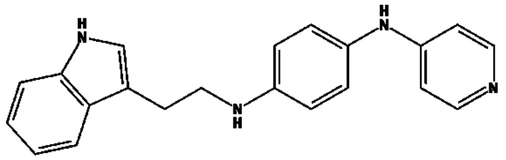
DB12558	AEE788	Multiple-receptor tyrosine kinase inhibitor (EGFR, VEGF, HER)	2J6M	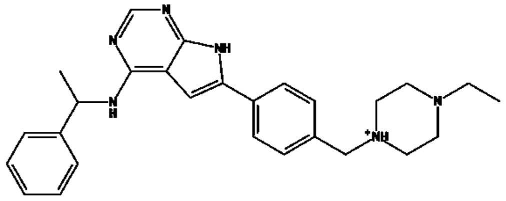

DB12882	Ombrabulin<br><br>(AVE8062)	Microtubule-destabilising agent, a combretastatin derivative that reached phase 3 clinical trial		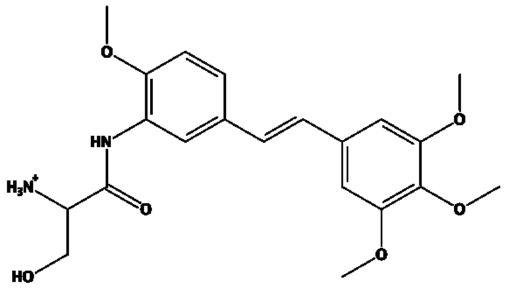
DB13248	Phthalylsulfathiazole	Sulfonamide antibacterial indicated in the treatment of gastrointestinal infections		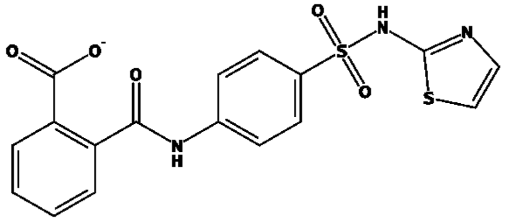
DB13948	25CN-NBOH	Selective serotonin receptor agonist (5-HT2A)	6WHA	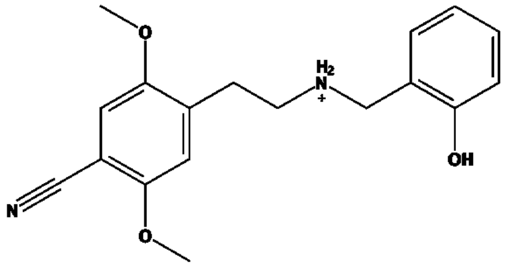
DB16236	Mitapivat<br>(AG-348)	Pyruvate kinase activator approved under the name of PYRUKYND for the treatment of hemolytic anemia caused by pyruvate kinase deficiency		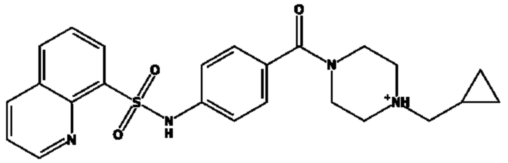
